# Safranal-loaded gold nanoparticles alleviate hepatocellular carcinoma via targeting the Wnt/β-catenin pathway

**DOI:** 10.1007/s12672-025-02447-w

**Published:** 2025-05-19

**Authors:** Yara A. Samra, Al Shaima G. Abd El Salam, Amr M. Abdelghany, Mamdouh M. El-Shishtawy

**Affiliations:** 1 Department of Biochemistry, Faculty of Pharmacy, Mansoura University, P.O. Box 35516, Mansoura, 35516 Egypt; 2 Department of Spectroscopy, Physics Division, National Research Center, Giza, 12311 Egypt; 3 Basic Science Department, Horus University, Coastal Road, New Damietta, Egypt

**Keywords:** Hepatocellular carcinoma, β-catenin, Safranal, Doxorubicin, Gold nanoparticles

## Abstract

**Background:**

The Wnt/β-catenin pathway is frequently activated in hepatocellular carcinoma (HCC); thus, it is considered a potential target for novel therapies. Safranal (SAF), a natural product, is reputed for its antitumor and antioxidant activities. Gold nanoparticles (AuNPs) exhibit unique physicochemical properties, they can carry and transport drugs to the tumor as they can passively accumulate within the tumor. The current study aims to evaluate SAF and SAF-AuNPs antitumor effect in HCC model via targeting the Wnt pathway and to evaluate the ability of SAF-AuNPs and Doxorubicin-gold nanoparticles (DOX-AuNPs) in ameliorating DOX chemo-resistance in HCC and enhancing its therapeutic index to reduce unwanted side effects.

**Results:**

SAF significantly attenuated the Wnt/β-catenin pathway, which down-regulated the proliferation and tumor angiogenesis. SAF decreased significantly Wnt-3a, β-catenin, Cyclin D1 VEGF and MMP-9. Developing SAF-AuNPs enhanced the antitumor activity of SAF against HCC. Furthermore, SAF-AuNPs enhanced DOX-AuNPs antitumor activity and lowered multi-drug resistance (MDR) protein level, which attenuates DOX chemo-resistance.

**Conclusions:**

We conclude that SAF and SAF-AuNPs are promising treatments for HCC. They have promising antitumor activity in addition to the ability to attenuate DOX chemo-resistance, so, the desired therapeutic effect may be obtained with minor doses and lowering the side effects.

**Supplementary Information:**

The online version contains supplementary material available at 10.1007/s12672-025-02447-w.

## Background

Among all the reasons for cancer-related death, hepatocellular carcinoma (HCC) is considered the fourth most prevalent cause of cancer-related death globally [1]. Mostly, HCC patients are asymptomatic, and they are diagnosed at late stages when surgical treatment is no longer suitable with limited treatment options. So, novel therapies for HCC should be found [2]. There were various signaling pathways activated in HCC such as the Wnt/β-catenin. It is activated in fifty percent of HCC tissues [3]. It regulates numerous cellular mechanisms that are implicated in HCC initiation, growth, survival, migration, differentiation, and apoptosis; thus, it is a potential target for novel molecular therapies [4].

The aberrant activation of the Wnt/β-catenin signaling pathway which occurs via deregulations of the pathway elements either by mutation or the up-/down-regulation plays a crucial role in the development of HCC [5–7].

Wnt/β-catenin signaling is activated via binding of the Wnt to the frizzled receptor (Fz). As a consequence, the destruction complex is deactivated and leads to β-catenin phosphorylation by glycogen synthase kinase 3 (GSK3) and casein kinase 1 (CK1) and subsequent proteasomal degradation. Consequently, accumulated non-phosphorylated β-catenin enters the nucleus and leads to an increase in vascular endothelial growth factor (VEGF), matrix metalloproteinase-9 (MMP-9), cyclin D1 and Multidrug resistance (MDR) [2, 7–10].

Doxorubicin (DOX) is a chemotherapy used to treat solid tumors and soft tissue cancers. It is commonly used in treatment of HCC [11, 12]. However, cardio-toxicity and the development of cancer chemo-resistance are the major limitations to its therapeutic effect, so, different strategies have been tried to overcome those limitations but the ability of these therapies to protect the heart from DOX-induced damage was limited [11, 13]. Thus, there is an imperative need to search for novel and safe strategies for HCC treatment.

Safranal (SAF) is an organic compound isolated from the stigmas of the flower of Crocus sativus (saffron). It is the major coloring constituent of saffron and is responsible for its special odor [14]. Previous studies have shown that SAF possesses potent anti-cancer, anti-inflammatory, and antioxidant effects [15–17]. Furthermore, it is characterized by selective toxicity against tumor cells and non-existent toxicity against normal cells [18].

Nanoparticles have risen as modern carriers of anti-cancer drugs. Gold nanoparticles (AuNPs) display interesting chemical and physical properties that encourage loading and make them able to be used as carriers for transporting the drug to the targeted sites. Their biocompatibility and high affinity for huge biomolecules such as drugs, proteins, chemicals, and DNA, further aid drug targeting and delivery [19, 20]. Moreover, they can pass through physiological barriers and passively accumulate within tumor sites due to their enhanced permeability and retention (EPR) effect [21, 22].

In this study, we prepared DOX as doxorubicin-gold nanoparticles (Dox-AuNPs), as AuNPs can act as idle carriers for DOX due to their inborn responsiveness to tumor cells. Thus, the specified therapeutic effect may be achieved with fewer doses and diminishing the related side effects. Also, we prepared SAF as safranal-gold nanoparticles (SAF-AuNPs) to improve their possible anti-tumor effect against HCC.

Therefore, the current study aimed to evaluate SAF and SAF-AuNPs antitumor effect in HCC model in rats, via altering the Wnt/β-catenin pathway. Also, we investigated SAF and SAF-AuNPs antioxidant activity in HCC rats. In addition, we evaluated the ability of the combination of SAF-AuNPs and DOX-AuNPs to ameliorate DOX chemo-resistance in HCC-rat model.

## Materials and methods

### Materials

TAA, 99% purity; Tetrachloroauric (III) acid (HAuCl_4_.3H_2_O), and SAF were obtained from Sigma Aldrich (MO, USA). DOX-HCl (Adricin) was obtained from EIMC United Pharmaceuticals (Badr City, Egypt).

### Animals

Ninety Male Sprague Dawley rats weighing 200–250 g were used in the present study. All animals were housed at 25 ± 2 °C and 12 h light/dark cycle system. Animals were provided with free access to water and nourishment. The experimental protocol was approved by the ethical committee of Faculty of Pharmacy, Mansoura University, Egypt (approval code: 2022-11) according to the “Principles of Laboratory Animal Care” (NIH publication No. 85-23, revised 1985).

All experiments were performed in accordance with relevant guidelines and regulations in accordance with The ARRIVE guidelines 2.0 [23].

*Accordance* We confirm that all experiments in this study were performed in accordance with the relevant guidelines and regulations.

*Arrive* All the procedure of the study is followed by the ARRIVE guidelines.

### Preparation of SAF-AuNPs and Dox-AuNPs

The small-size AuNPs were first synthesized via the green synthesis method. Menthe Piperita was collected from Mansoura University Garden. Fresh plant leaves were rinsed and cleaned several times using de-ionized water to avoid any metallic contamination. Menthe extract was prepared as previously reported [24, 25]. Tetrachloroauric acid was used during the synthesis process.

**Fig. 1:**
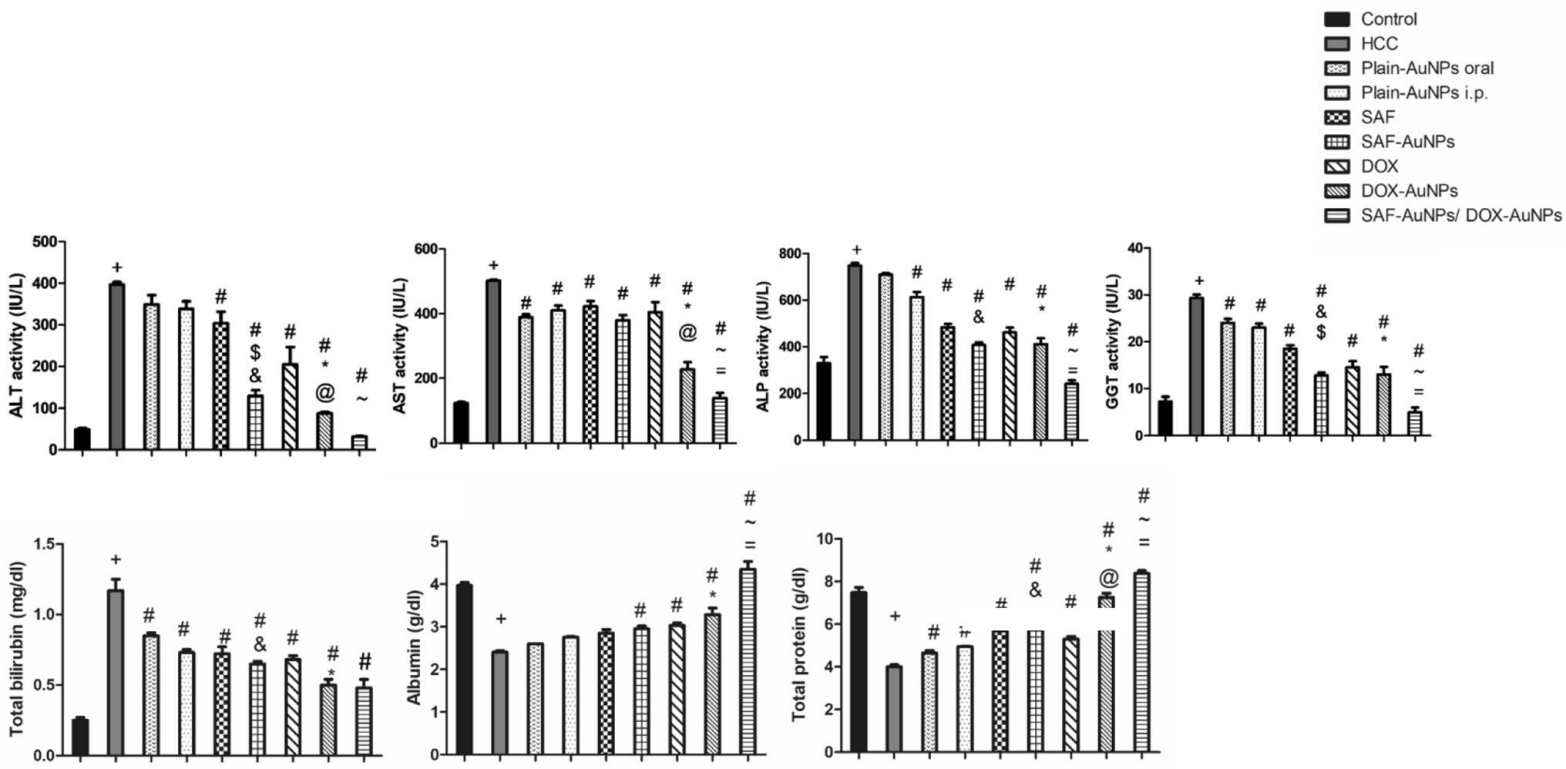
Effect of SAF, DOX, their AuNPs and their AuNPs combination on liver function parameters. Results were presented as mean ± SEM. N = 8 rats per group. ^+^p < 0.05 vs. control group, ^#^p < 0.05 vs. HCC group, ^*^p < 0.05 vs. Plain-AuNPs i.p. group, ^&^p < 0.05 vs. Plain-AuNPs oral group, ^$^p < 0.05 vs. SAF group, ^@^p < 0.05 vs. DOX group, ^~^p < 0.05 vs. SAF-AuNPs group, ^=^p < 0.05, vs. DOX-AuNPs group

**Fig. 2:**
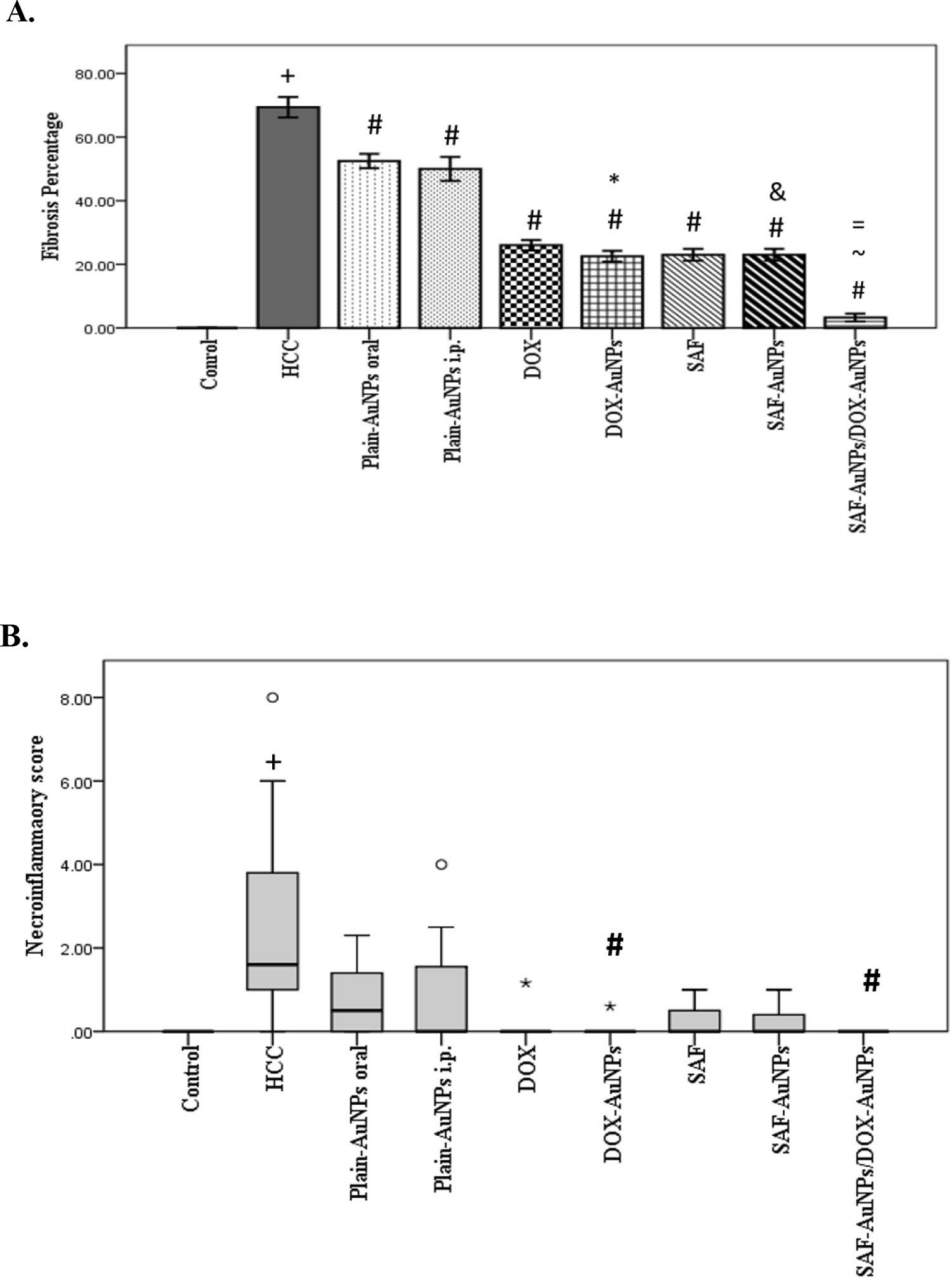
SAF-AuNPs/DOX-AuNPs combination therapy decreased fibrosis percentage (**a**) and necroinflammatory score (**b**) in liver. Fibrosis results were presented as mean ± SEM and necroinflammatory results were presented as median and range. N = 8 rats per group. ^+^p < 0.05 vs. control group, ^#^p < 0.05 vs. HCC group, ^*^p < 0.05 vs. Plain-AuNPs i.p. group, ^&^p < 0.05 vs. Plain-AuNPs oral group, ^~^p < 0.05 vs. SAF-AuNPs group, ^=^p < 0.05 vs. DOX-AuNPs group

**Fig. 3:**
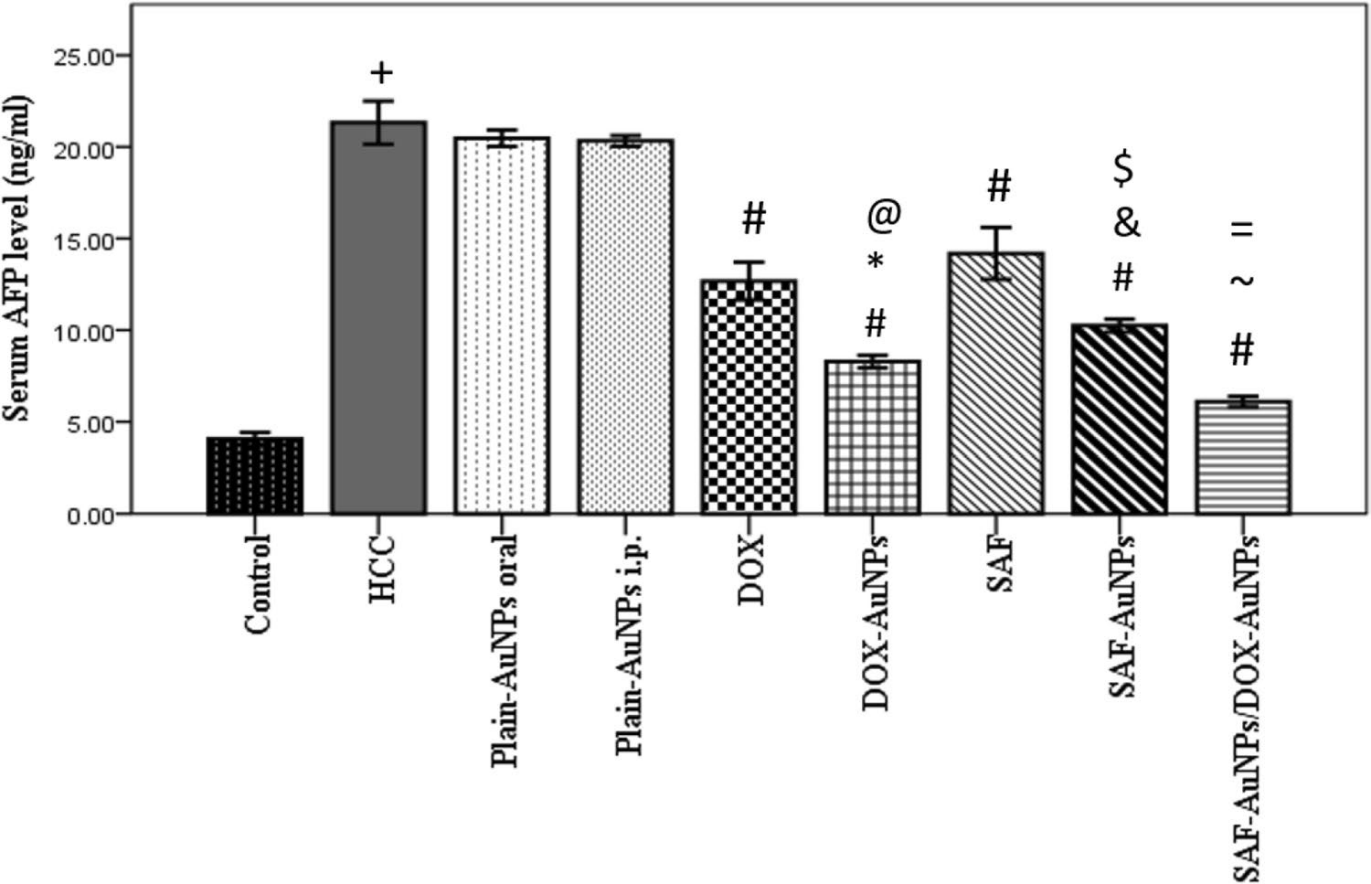
Effect of SAF-AuNPs/DOX-AuNPs combination therapy on serum AFP. Results were expressed as mean ± SEM. n = 8 rats per group. ^+^p < 0.05 vs. control group, ^#^p < 0.05 vs. HCC group, ^*^p < 0.05 vs. Plain-AuNPs i.p. group, ^&^p < 0.05 vs. Plain-AuNPs oral group, ^$^p < 0.05 vs. SAF group, ^@^p < 0.05 vs. DOX group, ^~^p < 0.05 vs. SAF-AuNPs group, ^=^p < 0.05, vs. DOX-AuNPs group

5 ml of the fresh plant extract was added drop by drop to 100 ml of 1 mM Tetrachloroauric acid until the color changed from pale brownish to purple reddish. The color change indicated the formation of AuNPs that was confirmed via Ultraviolet–visible (UV–vis) spectroscopy, obtained spectral data were characterized by two main absorption bands centered at 210 and 533 nm (Fig. 1S). The strong UV absorption band at 210 nm correlated with the residuals of plant extract used for the synthesis of gold even present in ppm level, while the strong visible band centered at about 533 nm is known to characterize the SPR process of synthesized AuNPs (Fig. 1S). Also, the crystalline nature of the synthesized AuNPs was observed through the appearance of characteristic sharp bands in the X-ray diffraction (XRD) (Fig. 2S). Particle size and morphology were measured using transmission electron microscopy (TEM) supported with selected area electron diffraction (SAED). Transmission electron microscopic (TEM) investigation of synthesized AuNPs revealed a uniform distribution of distorted spherical nanoparticles of average size ranging between 9 and 18 nm. (Fig. 3S), in addition to the DLS using the Malvern Panalytical instrument.

**Fig. 4:**
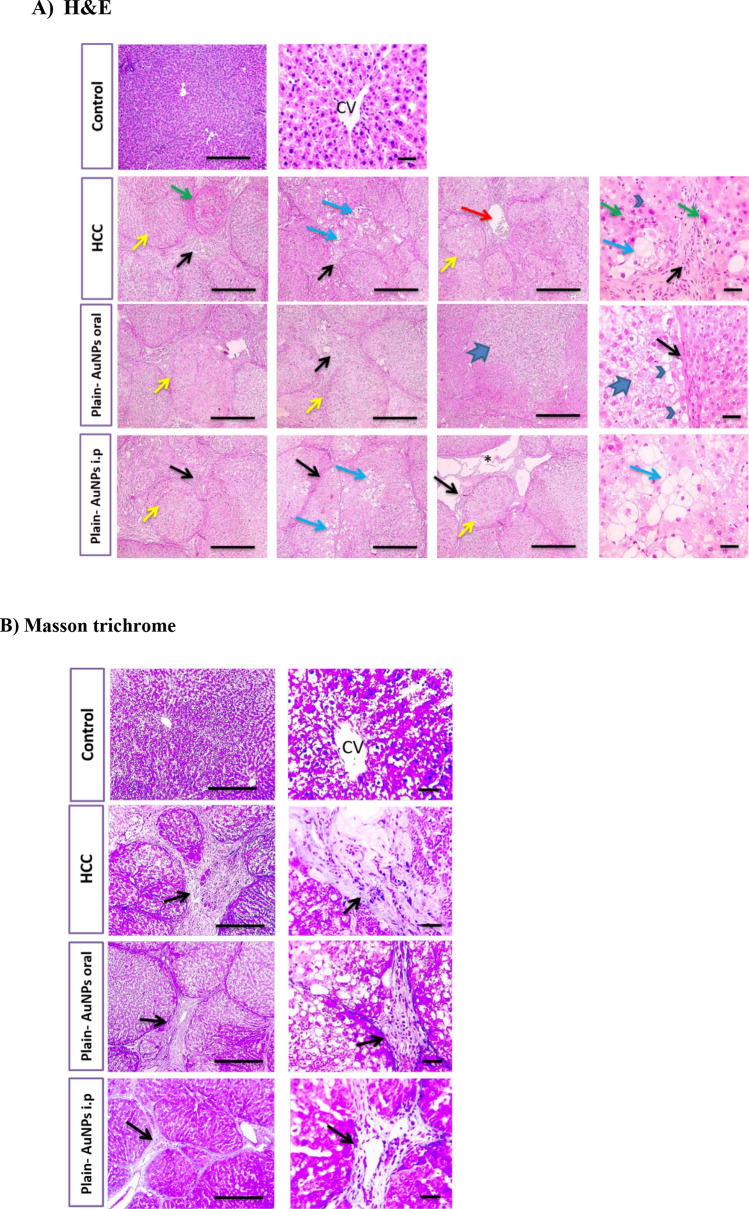
Histopathological examination of liver tissue in control, HCC, Plain-AuNPs oral, and Plain- AuNPs i.p. groups. **a** H&E-stained liver sections, Control group presented normal hepatic cords with normal portal areas and sinusoids. However, HCC group exhibited disrupted parenchymal structure and widespread fibrosis (black arrows) scored 6, also showed inflammatory cells infiltration such as hemosiderin-laden macrophages and showed congested blood vessels (red arrows). Hepatocytes are present in solid nodules (yellow arrows) with micro-vesicular degeneration (arrowheads), ballooning degeneration (blue arrows) and necrosis (green arrows). Plain-AuNPs oral and Plain-AuNPs i.p. groups showed the same changes in addition to hydropic degeneration (thick arrows) and edema (asterisk). **b** Masson’s trichome-stained liver sections, control group showed no collagen deposition. However, HCC group displayed excessive collagen deposition with blue color (black arrows). Plain-AuNPs oral and Plain-AuNPs i.p. groups showed a minor reduction in collagen deposition (black arrows). n = 8 rats per group. Low magnification X: 100 bar 100 and high magnification X: 400 bar 50

Also, zeta size and zeta potential were performed using Malvern zetasizer Nabo-ZS90 (Fig. 4S). The zeta size and zeta potential measurements of the green-synthesized gold nanoparticles (AuNPs) provide critical insights into their physical and colloidal properties. The average particle size at the maximum peak of approximately 73.5 nm indicates that the synthesized AuNPs are relatively uniform and fall within the nanoscale range, which is consistent with typical green synthesis methods that often yield particles in this size range. The zeta potential peak at around − 17 mV suggests that the nanoparticles possess a moderate negative surface charge. This negative charge is likely due to the capping agents or stabilizing molecules derived from the green synthesis process, such as plant extracts or biomolecules. While a zeta potential of − 17 mV indicates colloidal stability. However, the stability of these nanoparticles could still be maintained through steric stabilization provided by the capping agents.

For SAF and DOX loading, SAF and DOX were added to AuNP (380 ppm) solution with a ratio of 1:4. The mixtures were sonicated for 20 min. The volume of the SAF-AuNPs mixture was expanded to 1 ml by normal saline.

### Experimental design

TAA was dissolved in normal saline and (200 mg/kg) was injected in rats intra-peritoneal (i.p.) twice weekly for 16 weeks [26] to induce HCC. After 16 weeks, eighteen rats died.

After that, rats were equally distributed into nine groups. Each group contains eight animals as follows:

*Control group* Normal rats without treatment. *HCC group* rats injected by TAA for induction of HCC. *SAF group* rats treated with oral SAF at (25 mg/kg) per day for seven weeks after induction of HCC [27]. *SAF-AuNPs group* rats received SAF-AuNP mixture orally once daily; for seven weeks following induction of HCC. *DOX group* rats received i.p. DOX (1 mg/kg) twice/week for 7 weeks after induction of HCC [28]. *DOX-AuNPs group* rats received DOX-AuNPs mixture; i.p. twice/week for seven weeks following the induction of HCC. *SAF-AuNPs/DOX-AuNPs group* rats received a combination mixture of SAF-AuNPs and DOX-AuNPs for 7 weeks after induction of HCC. *Plain-AuNPs oral group* rats received AuNPs (380 ppm) solution orally once daily for seven weeks following induction of HCC. *Plain-AuNPs i.p. group* rats received AuNPs (380 ppm) solution; i.p. daily for 7 weeks following the induction of HCC.

### Sample collection

The occurrence of HCC was confirmed by assessment of α-fetoprotein (AFP) serum level and histopathological analysis. Blood samples were collected by Retro-orbital puncture and centrifuged. Serum was collected and stored at − 80 °C for further assessment of liver function tests [total protein, albumin, bilirubin levels and aspartate aminotransferase (AST), alanine aminotransferase (ALT), gamma-glutamyl transferase (GGT) and alkaline phosphatase (ALP) activities]. Then, rats were sacrificed, and the liver was isolated.

There will be no surgical procedures or post-surgical procedures. There’s no induction of pain during the experiment so there is no need for painkillers, but if needed at any point the animals will be given ketoprofen (3 mg/kg/IM). In Euthanasia animals will be deeply anesthetized by secobarbital 50 mg/kg/IP.

Part of the liver was fixed in phosphate-buffered formalin 10% (pH 7.2) and used for immunohistochemical and histopathological experiments. The other part of the liver was homogenized in cold phosphate-buffered saline (PBS) (pH 7.4). Then this mix was centrifuged and stored at − 80 °C to be used for colorimetric detection of hepatic glutathione (GSH) and malondialdehyde (MDA). Also, used for ELISA detection of hepatic Wnt-3a, Cyclin D, β-catenin, MDR and MMP-9 using kits from Bioassay Technology Laboratory. Also, ELISA Kit from Atlas Medical, Germany was used to measure serum AFP.

### Histopathological examination of liver

Formalin-fixed liver tissues were embedded into paraffin blocks. To assess necroinflammatory grades, 5 μm-thickness sectors were divided and stained with hematoxylin and eosin (H&E) stain. Histopathological changes were detected using Ishak et al. modified Histology activity index (HAI) system [29].

To quantify fibrosis percentage, hepatic portions were stained with Masson's trichrome. Histopathological changes were detected and photographed using a digital camera seated on a BX51 Olympus optical microscope (Olympus Corporation, Tokyo, Japan). NIH Image software was used to assess collagenous areas.

### Immunohistochemistry (IHC)

The immunohistochemical staining procedures of VEGF were done according to Saber et al. [30]. Sections were de-waxed and immersed in a solution of 0.05 M citrate buffer, pH 6.8, for antigen retrieval. These sections were then treated with 0.3% H_2_O_2_ and protein block. After that, they were incubated with polyclonal rabbit anti-VEGF antibody (Thermo Fisher Scientific, Cat. No. PA5-16754, dilution 1/100), after rinsing with PBS, they were incubated with a goat anti-rabbit secondary antibody (Cat. No. K4003, EnVision + ^™^ System Horseradish Peroxidase Labelled Pomer; Dako) for 30 min at room temperature. Slides were visualized with a 3,3′-Diaminobenzidine (DAB) kit and ultimately stained with Mayer’s hematoxylin as a counterstain. Also, immunohistochemical staining was done for Cyclin D1 and MMP-9. The staining intensity was assessed by Image J analysis software (NIH, USA).

### Statistical analysis

Results were analyzed by one-way analysis of variance (ANOVA) followed by Tukey's post-hoc test. A non-parametric Kruskal–Wallis test followed by Dunn’s post-hoc test was used to investigate necro-inflammatory scoring results. Data were expressed as mean ± SEM. Necro-inflammatory scoring was expressed as median and range. Statistical analysis was done by SPSS Statistics version 20. Statistical significance was p < 0.05.

## Results

### Effect of SAF, DOX, and their AuNPs on liver function

SAF-AuNPs significantly improved liver function. Serum activities of AST, ALT, ALP, GGT, and bilirubin level were significantly decreased by 67.54%, 24.4%, 45.56% 56.26%, and 44.3%, respectively, with p < 0.001, compared to HCC and ALT and GGT activities were extensively decreased by 57.58% (p < 0.001) and 30.65% (p < 0.01), respectively, compared to SAF. Also, SAF-AuNPs drastically increased albumin and total protein levels by 20.83% (p < 0.05) and 43.25% (p < 0.001), respectively than the HCC group.

DOX-AuNPs revealed more marked improvement in liver functions than DOX. DOX-AuNPs significantly decreased ALT, AST, ALP, GGT and bilirubin by 77.86%, 54.75%, 45.09%, 55.68% and 57.16% (p < 0.001), respectively, compared to HCC. Also, DOX-AuNPs significantly reduced ALT and AST activities by 57.09% (p < 0.01) and 43.9% (p < 0.001), respectively, compared to DOX. In addition, DOX-AuNPs revealed 36.67% (p < 0.001) and 81.25% (p < 0.001) significant elevation in albumin and total protein levels, respectively, as compared to HCC group and 36.79% (p < 0.001) significant elevation in total protein level as compared to DOX group.

Interestingly, SAF-AuNPs/DOX-AuNPs combination showed significant improvement in all parameters of liver function tests compared to the HCC group. SAF-AuNPs/DOX-AuNPs combination significantly decreased AST (p < 0.05), ALP, and GGT (p < 0.001) serum activities and increased albumin (p < 0.001) and total protein (p < 0.05) compared to DOX-AuNPs group. Furthermore, SAF-AuNPs/DOX-AuNPs group showed a non-significant difference from the control group in serum ALT, AST, ALP, GGT activities, albumin, and total protein levels. Data is shown in Table 1 and Fig. 1.

**Table 1:** Effect of SAF, DOX, their AuNPs and their AuNPs combination on liver function parameters

Group	ALT activity (IU/L)	AST activity (IU/L)	ALP activity (IU/L)	GGT activity (IU/L)	Total bilirubin (mg/dl)	Albumin (g/dl)	Total protein (g/dl)
Control	49.00 ± 3.15	124.33 ± 3.97	329.83 ± 26.56	7.33 ± 0.989	0.25 ± 0.02	3.97 ± 0.07	7.48 ± 0.24
HCC	396.67 ± 6.39 ^+^	501.67 ± 3.66 ^+^	748.50 ± 10.957^+^	29.33 ± 0.760^+^	1.17 ± 0.08^+^	2.40 ± 0.04^+^	4.00 ± 0.10^+^
Plain-AuNPs oral	349.00 ± 22.36	388.50 ± 9.17^#^	710.00 ± 6.01	24.00 ± 0.894^#^	0.85 ± 0.02^#^	2.60 ± 0.00	4.65 ± 0.12^#^
Plain-AuNPs i.p	338.00 ± 18.34	409.50 ± 15.429^#^	613.00 ± 22.36^#^	23.00 ± 0.894 ^#^	0.73 ± 0.02^#^	2.75 ± 0.02	4.95 ± 0.02^#^
SAF	303.67 ± 27.8 ^#^	422.33 ± 16.28^#^	483.50 ± 15.108^#^	18.50 ± 0.764^#^	0.72 ± 0.05^#^	2.85 ± 0.08	5.68 ± 0.26^#^
SAF-AuNPs	128.83 ± 13.7^# & $^	379.17 ± 16.2^#^	407.50 ± 11.20^# &^	12.83 ± 0.601^#& $^	0.65 ± 0.02^# &^	2.95 ± 0.07^#^	5.73 ± 0.42^# &^
DOX	204.67 ± 41.95^#^	404.67 ± 30.99^#^	461.50 ± 20.813^#^	14.50 ± 1.335^#^	0.68 ± 0.03^#^	3.03 ± 0.06^#^	5.30 ± 0.12^#^
DOX-AuNPs	87.83 ± 3.50^# * @^	227.00 ± 22.675^# *@^	411.00 ± 25.343^# *^	13.00 ± 1.653^# *^	0.50 ± 0.04^# *^	3.28 ± 0.16^# *^	7.25 ± 0.20 ^# * @^
SAF-AuNPs/ DOX-AuNPs	31.67 ± 1.50^# ~^	139.50 ± 15.05^# ~ =^	240.83 ± 14.961^# ~ =^	5.00 ± 1.065^# ~ =^	0.48 ± 0.06^#^	4.35 ± 0.18^# ~ =^	8.38 ± 0.13^# ~ =^

Data were expressed as mean ± SEM. n = 8 rats per group

^**+**^p < 0.05 vs. control group. ^**#**^p < 0.05 vs. HCC group. ^*^p < 0.05 vs. Plain- AuNPs i.p. group. ^**&**^p < 0.05 vs. Plain- AuNPs oral group. ^**@**^p < 0.05 vs. DOX group. ^**=**^p < 0.05 vs. DOX- AuNPs group. ^**$**^p < 0.05 vs. SAF group. ^**~**^p < 0.05 vs. SAF- AuNPs group

### SAF-AuNPs/DOX-AuNPs combination therapy decreased fibrosis and necroinflammation in liver homogenates

HCC group revealed a striking increase in fibrosis than the control group (p < 0.001). Oral Plain-AuNPs and SAF significantly decreased the fibrosis by 24.39% (p < 0.001) and 66.86% (p < 0.001), respectively, than the HCC group. SAF-AuNPs group showed a more marked decrease in fibrosis (74.54%; p < 0.001) than the HCC group. Intra-peritoneal Plain-AuNPs and DOX groups significantly lowered the fibrosis by 27.95% (p < 0.001) and 62.54% (p < 0.001), respectively, than the HCC group, while DOX-AuNPs group significantly lowered the percentage of fibrosis by 67.54% (p < 0.001). In addition, the SAF-AuNPs group significantly reduced fibrosis (p < 0.001) than the Plain-AuNPs oral group. Also, the DOX-AuNPs group revealed a significant decrease in fibrosis (p < 0.001) than the Plain-AuNPs i.p. group (Fig. 2a).

Moreover, SAF-AuNPs/DOX-AuNPs combination significantly decreased fibrosis compared to the HCC group (p < 0.001). Also, this combination significantly decreased the fibrosis compared to either SAF-AuNPs (81.51% and p < 0.001) or DOX-AuNPs (85.5% and p < 0.001) (Fig. 2a).

In parallel, HCC produced a higher rise in necroinflammatory scores than the control group (p < 0.01). DOX-AuNPs group exhibited a significant decrease in necroinflammatory scores (p < 0.05) than the HCC group. Moreover, SAF-AuNPs/DOX-AuNPs combination significantly decreased necroinflammatory scores (p < 0.01) than the HCC group (Fig. 2b).

### SAF-AuNPs debilitate HCC and augment DOX-AuNPs antitumor activity

HCC rats showed a significant elevation in serum AFP level (p < 0.001) than the control group. Both Plain-AuNPs oral and Plain-AuNPs i.p. revealed a non-significant lowering in serum AFP level. However, SAF and DOX significantly decreased serum AFP levels compared to HCC (p < 0.001). SAF-AuNPs significantly lowered the AFP level by 51.09% (p < 0.001) as compared to HCC, and by 27.72% (p < 0.001) as compared to SAF. Moreover, DOX-AuNPs significantly decreased the AFP level by 61.21% (p < 0.001) than HCC, and by 34.70% (p < 0.001) than DOX. Also, SAF-AuNPs and DOX-AuNPs significantly decreased serum AFP than Plain-AuNPs oral and Plain-AuNPs i.p. (p < 0.001) (Fig. 3).

Additionally, SAF-AuNPs/DOX-AuNPs combination significantly reduced serum AFP than HCC group (p < 0.001). Also, SAF-AuNPs/ DOX-AuNPs combination therapy revealed a more significant lowering in serum AFP level by 68.03%(p < 0.001) as compared to SAF-AuNPs and by 35.73% (p < 0.05) as compared to DOX-AuNPs (Fig. 3).

In parallel, normal control group liver sections stained with H&E showed a normal arrangement of liver architecture and Masson trichrome showed no collagen deposition. HCC group showed disrupted parenchymal structure and widespread fibrosis accompanying inflammatory cells infiltration with excessive collagen deposition dividing hepatic lobules into separate large tumor nodules. Plain-AuNPs oral and Plain-AuNPs i.p. groups showed similar changes in the degree of disturbed liver architecture as the HCC group with a slight reduction in collagen deposition (Fig. 4a, b).

**Fig. 5:**
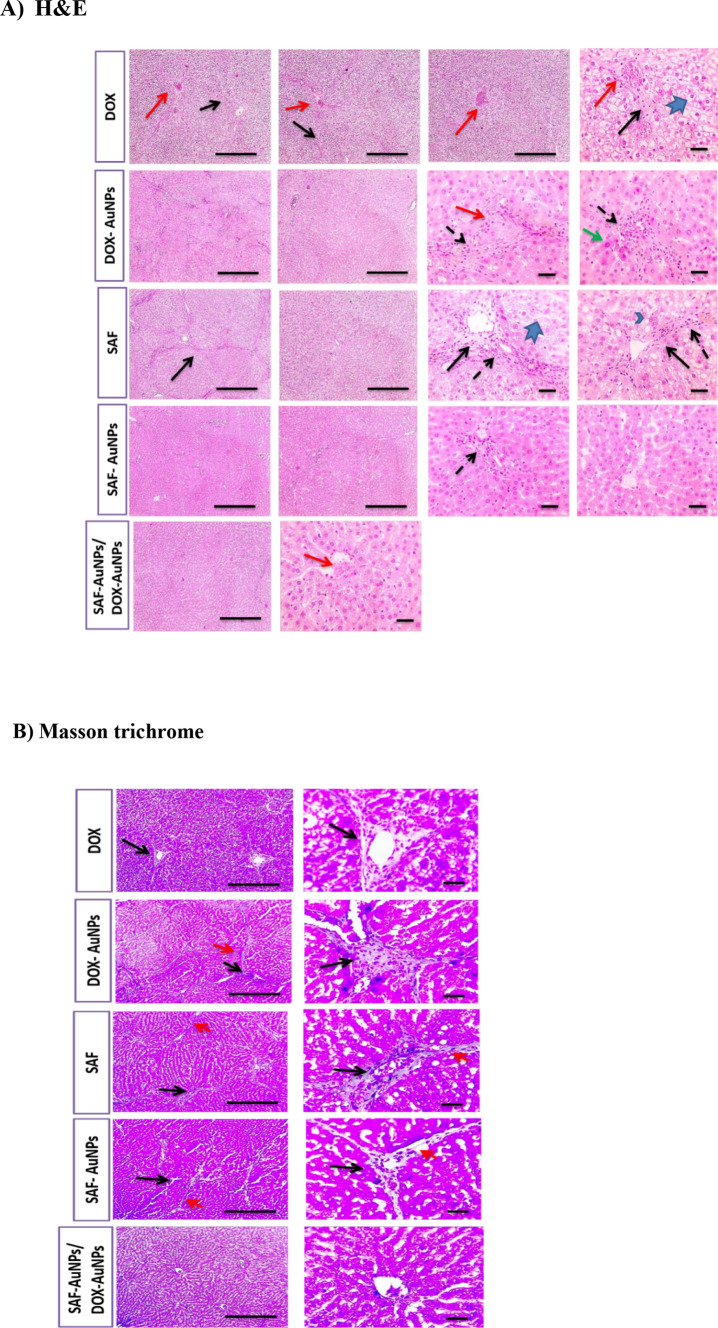
Histopathological examination of liver tissue of DOX, DOX-AuNPs, SAF, SAF-AuNPs, and SAF-AuNPs/DOX-AuNPs groups. a) H&E-stained liver sections, DOX group showed slightly better hepatic parenchymal structure with portal fibrosis (black arrows) scored 3 and infiltration of inflammatory cells such as hemosiderin-laden macrophages and showed congested blood vessels (red arrows). Hepatocytes suffered from hydropic degeneration (thick arrows). DOX-AuNPs group showed partially improved hepatic parenchymal structure with congested blood vessels (red arrows) surrounded by infiltrated inflammatory cells that include hemosiderin-laden macrophages (dashed arrows). Few necrotic hepatocytes are present (green arrows). SAF group showed slightly enhanced hepatic parenchymal structure with portal fibrosis (black arrows) scored 4 and inflammatory cells infiltration such as hemosiderin-laden macrophages (dashed arrows). Hepatocytes suffered from macro-vesicular degeneration (arrowheads) to hydropic degeneration (thick arrows). SAF-AuNPs group showed intensely improved hepatic parenchymal structure with few inflammatory cells’ infiltration in portal areas (dashed arrows). SAF-AuNPs/DOX-AuNPs group showed restored hepatic parenchymal structure with very mild congestion (red arrows). **b** Masson’s trichome-stained liver sections, DOX group showed mild collagen deposition blue color (black arrows). DOX-AuNPs group showed mild collagen deposition blue color (black arrows) and thin collagen strands spreading from portal areas (red arrow). SAF and SAF-AuNPs groups showed slight perivascular collagen deposition (black arrows) with very thin collagen strands spreading from portal areas (red arrows). Hepatic sections from SAF-AuNPs/DOX-AuNPs group showed no collagen deposition. n = 8 rats per group. Low magnification X: 100 bar 100 and high magnification X: 400 bar 50

Hepatic sections from the SAF group showed a slight recovery in hepatic parenchymal structure compared to the HCC group, while the SAF-AuNPs group showed great improvement in hepatic parenchymal structure compared to the HCC group and was characterized by few inflammatory cells’ infiltration in portal areas. Both SAF and SAF-AuNPs groups showed mild perivascular collagen deposition. In addition, the DOX group showed a slight improvement in hepatic parenchymal structure, while the DOX-AuNPs group showed partial improvement of hepatic parenchymal structure characterized by few necrotic hepatocytes as compared to the HCC group. Both DOX and DOX-AuNPs groups showed mild perivascular collagen deposition. Moreover, SAF-AuNPs/DOX-AuNPs combination therapy showed restoration of hepatic parenchymal structure with very mild congestion and with no collagen deposition (Fig. 5a, b).

### Effect of SAF-AuNPs/ DOX-AuNPs combination therapy on hepatic Wnt-3a protein level

**Fig. 6:**
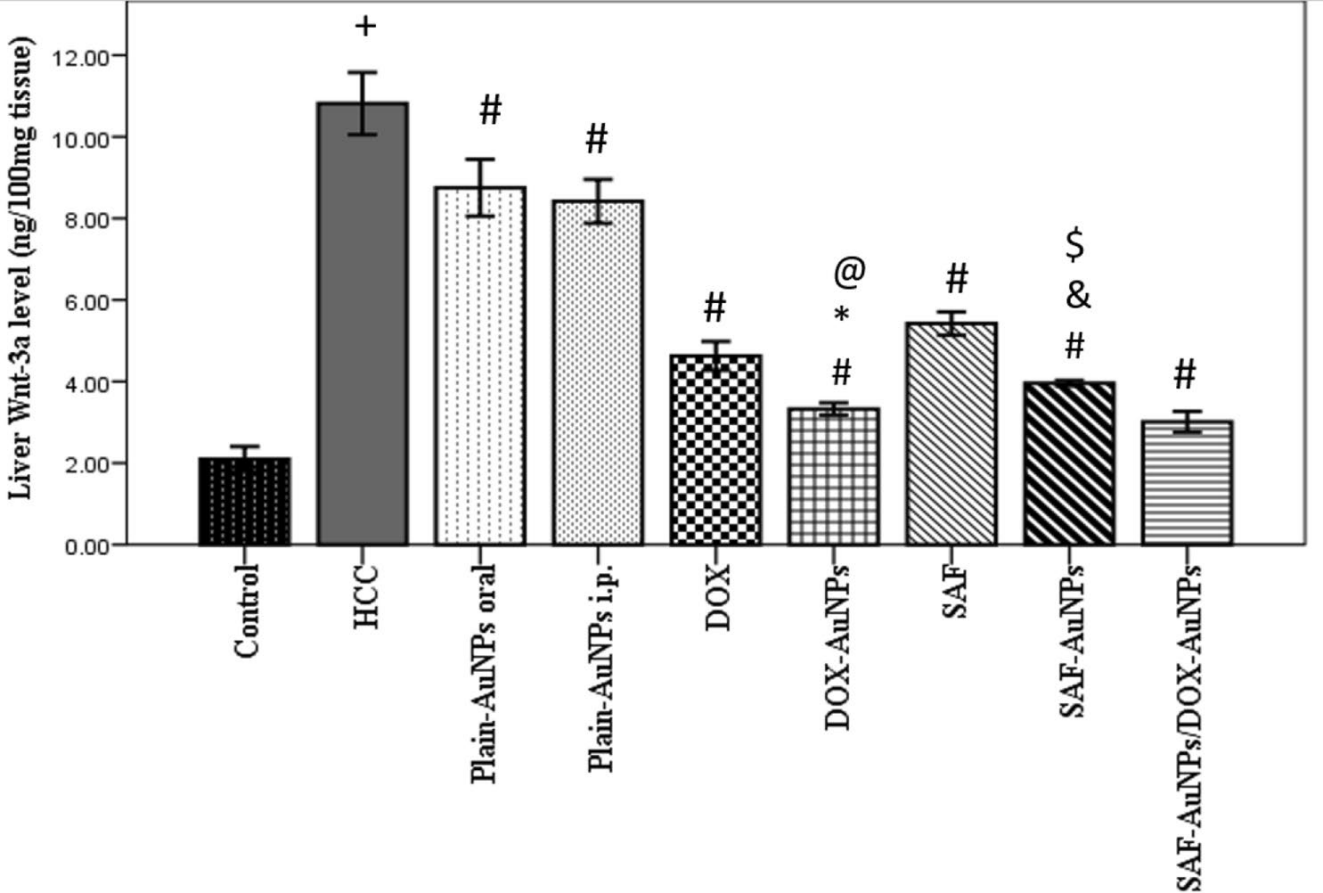
Effect of SAF-AuNPs/DOX-AuNPs combination therapy on hepatic Wnt-3a protein level. Results were presented as mean ± SEM. n = 8 rats per group. ^+^p < 0.05 vs. control group, ^#^p < 0.05 vs. HCC group, ^*^p < 0.05 vs. Plain-AuNPs i.p. group, ^&^p < 0.05 vs. Plain-AuNPs oral group, ^$^p < 0.05 vs. SAF group, ^@^p < 0.05 vs. DOX group

HCC group revealed a significant rise in hepatic Wnt-3a (p < 0.001) than the control group. Plain-AuNPs oral and SAF groups significantly decreased Wnt-3a level in the liver by 19.06% (p < 0.001) and 49.86% (P < 0.001), respectively, than HCC group. SAF-AuNPs showed a more marked decrease in Wnt-3a level by 63.37% (p < 0.001) than HCC group, and by 26.94% (p < 0.001) than SAF group. Plain-AuNPs i.p. and DOX groups significantly decreased hepatic Wnt-3a level by 22.11% (p < 0.001) and 57.17% (p < 0.001) than HCC group, while DOX-AuNPs significantly decreased Wnt-3a level by 69.2% (p < 0.001) compared to HCC, and by 28.08% (p < 0.01) compared to DOX. Also, SAF-AuNPs and DOX-AuNPs revealed a significant decrease in hepatic Wnt-3a level as compared to Plain-AuNPs oral and Plain-AuNPs i.p. (p < 0.001). Furthermore, SAF-AuNPs/DOX-AuNPs combination therapy showed a more significant decrease in Wnt-3a level in the liver by 72.16% (p < 0.001) as compared to the HCC group (Fig. 6).

### Effect of SAF-AuNPs/ DOX-AuNPs combination therapy on β-catenin protein level in liver

**Fig. 7:**
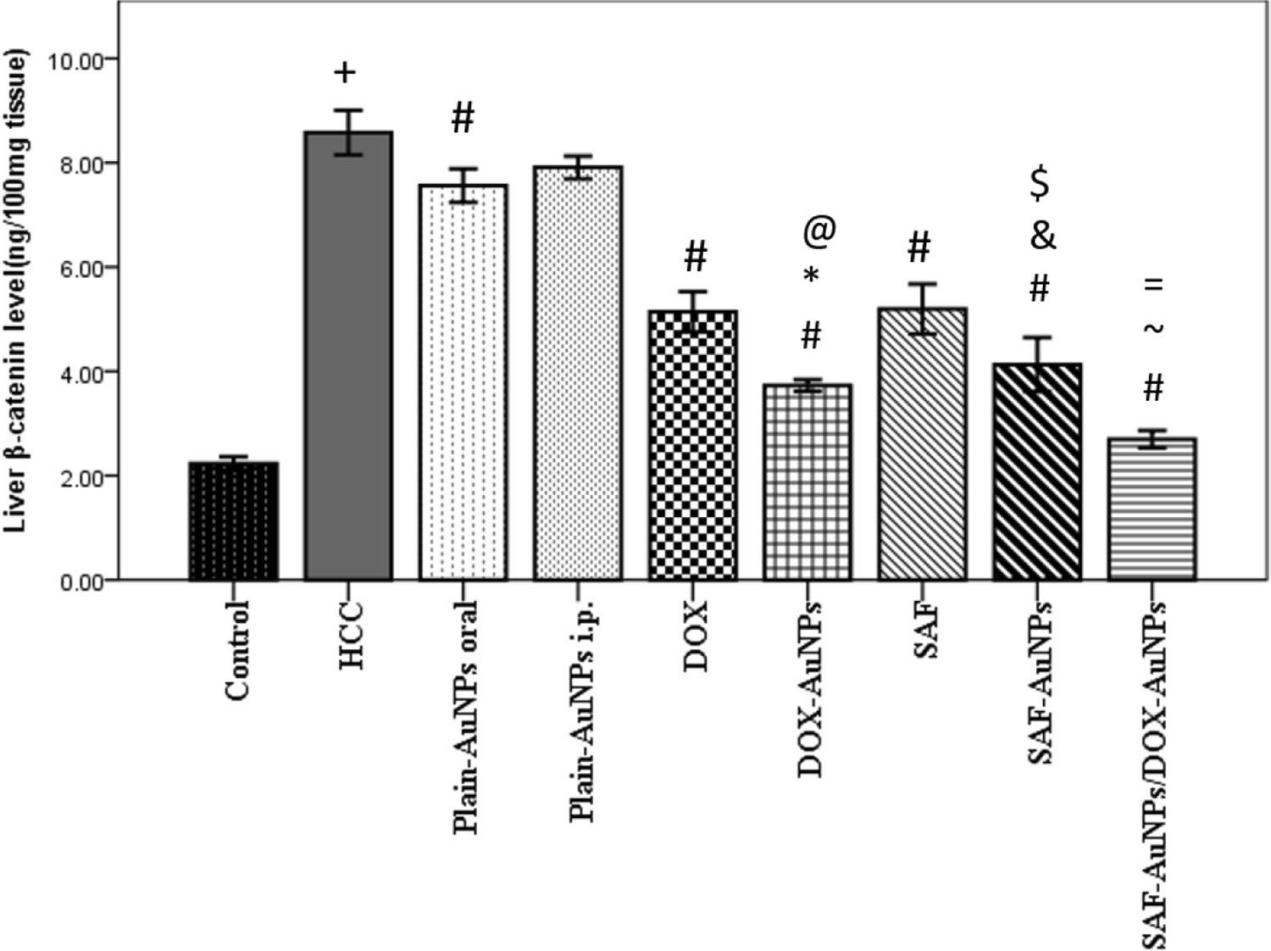
Effect of SAF-AuNPs/DOX-AuNPs combination therapy on hepatic β-catenin protein level. Results were presented as mean ± SEM. n = 8 rats per group. ^+^ p < 0.05 vs. control group, ^#^ p < 0.05 vs. HCC group, ^*^p < 0.05 vs. Plain-AuNPs i.p. group, ^&^p < 0.05 vs. Plain-AuNPs oral group, ^$^p < 0.05 vs. SAF group, ^@^p < 0.05 vs. DOX group, ^~^p < 0.05 vs. SAF-AuNPs group, ^=^p < 0.05 vs. DOX-AuNPs group

Β-catenin level was significantly raised in the liver of HCC group (p < 0.001) than in the control group. Plain-AuNPs oral and SAF groups significantly decreased β-catenin level by 11.89% (p < 0.01) and 39.51% (p < 0.001), respectively than HCC group. SAF-AuNPs highly decreased hepatic β-catenin level by 51.86% (p < 0.001) in comparison with HCC group, and by 20.42% (p < 0.01) in comparison with SAF group. Plain-AuNPs i.p. showed a non-significant decrease in β-catenin level in the liver compared to the HCC group, while DOX and DOX-AuNPs groups significantly decreased β-catenin level by 40.09% (p < 0.001) and 56.53% (p < 0.001) respectively than HCC group. Furthermore, the DOX-AuNPs group showed a more marked decrease in β-catenin level by 27.43% (p < 0.001) than the DOX group. SAF-AuNPs and DOX-AuNPs revealed a significant decrease in β-catenin level than Plain-AuNPs oral and Plain-AuNPs i.p. (p < 0.001). Moreover, SAF-AuNPs/DOX-AuNPs combination showed a significant decrease in β-catenin level than HCC group (p < 0.001). Also, SAF-AuNPs/DOX-AuNPs combination therapy revealed a significant decrease in β-catenin level (p < 0.001) than SAF-AuNPs and (p < 0.01) as compared to DOX-AuNPs (Fig. 7).

### Effect of SAF-AuNPs/ DOX-AuNPs combination therapy on hepatic MDR protein level

**Fig. 8:**
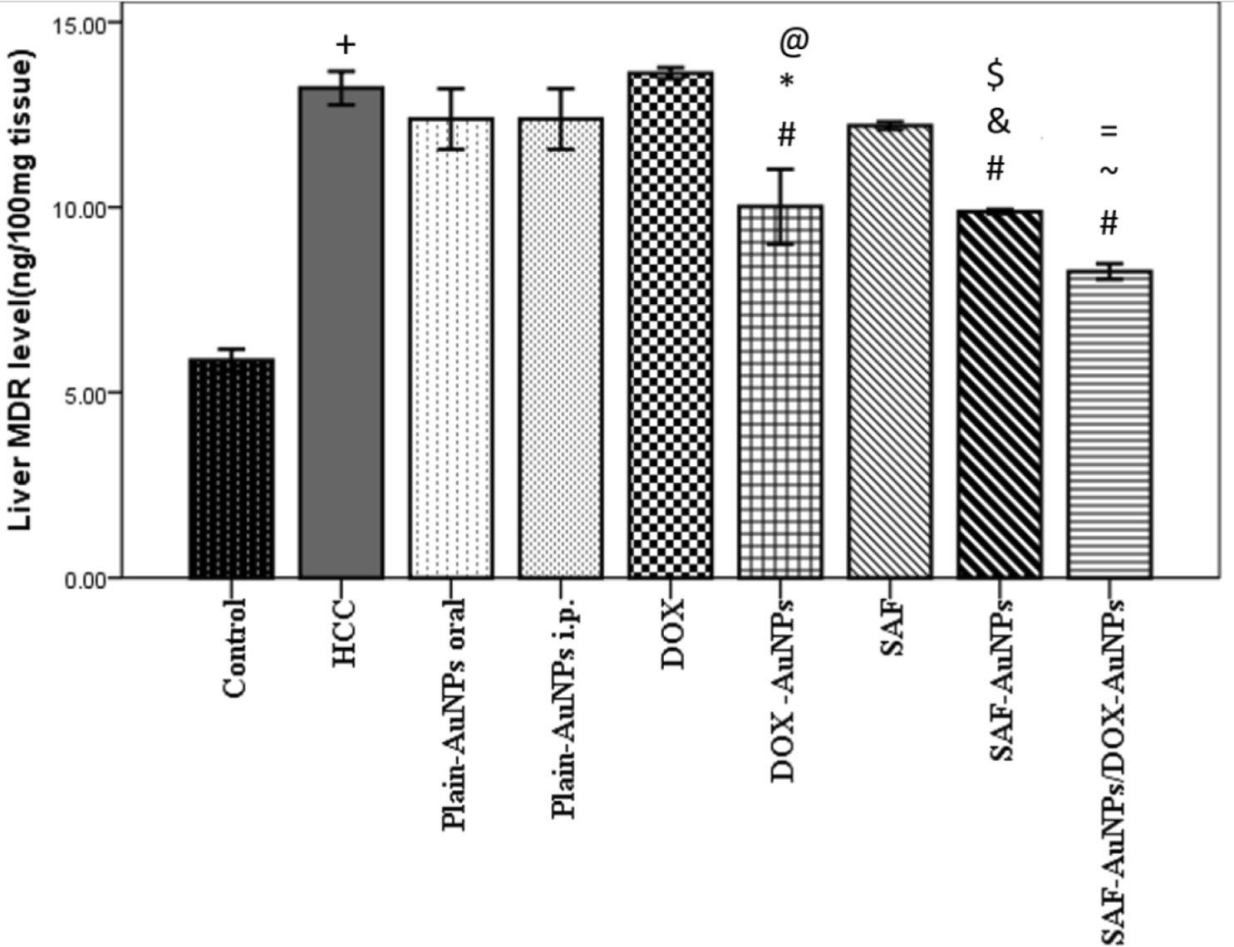
Effect of SAF-AuNPs/ DOX-AuNPs combination therapy on hepatic MDR protein level. Results were presented as mean ± SEM. n = 8 rats per group. ^+^p < 0.05 vs. control group, ^#^p < 0.05 vs. HCC group, ^*^p < 0.05 vs. Plain-AuNPs i.p. group, ^&^p < 0.05 vs. Plain- AuNPs oral group, ^$^p < 0.05 vs. SAF group ^@^p < 0.05 vs. DOX group, ^~^p < 0.05 vs. SAF-AuNPs group, ^=^p < 0.05 vs. DOX-AuNPs group

Hepatic MDR protein level showed a significant elevation in the HCC group (p < 0.001) than the control group. SAF-AuNPs group showed a marked reduction in MDR level by 25.26% (p < 0.001) than HCC group, and by 19.02% (p < 0.01) compared with the SAF group. DOX-AuNPs group showed a marked reduction in MDR level by 24.21% (p < 0.001) than HCC group, and by 26.43% (p < 0.001) than DOX group. Also, SAF-AuNPs and DOX-AuNPs revealed a significant decrease in hepatic MDR level as compared to Plain-AuNPs oral and Plain-AuNPs i.p. (p < 0.001). Moreover, SAF-AuNPs/DOX-AuNPs combination showed a significant decrease in MDR protein level than HCC group (p < 0.001) and (p < 0.01) as compared to SAF-AuNPs and DOX-AuNPs (Fig. 8).

### Effect of SAF-AuNPs/DOX-AuNPs combination therapy on hepatic Cyclin D1 protein level

**Fig. 9:**
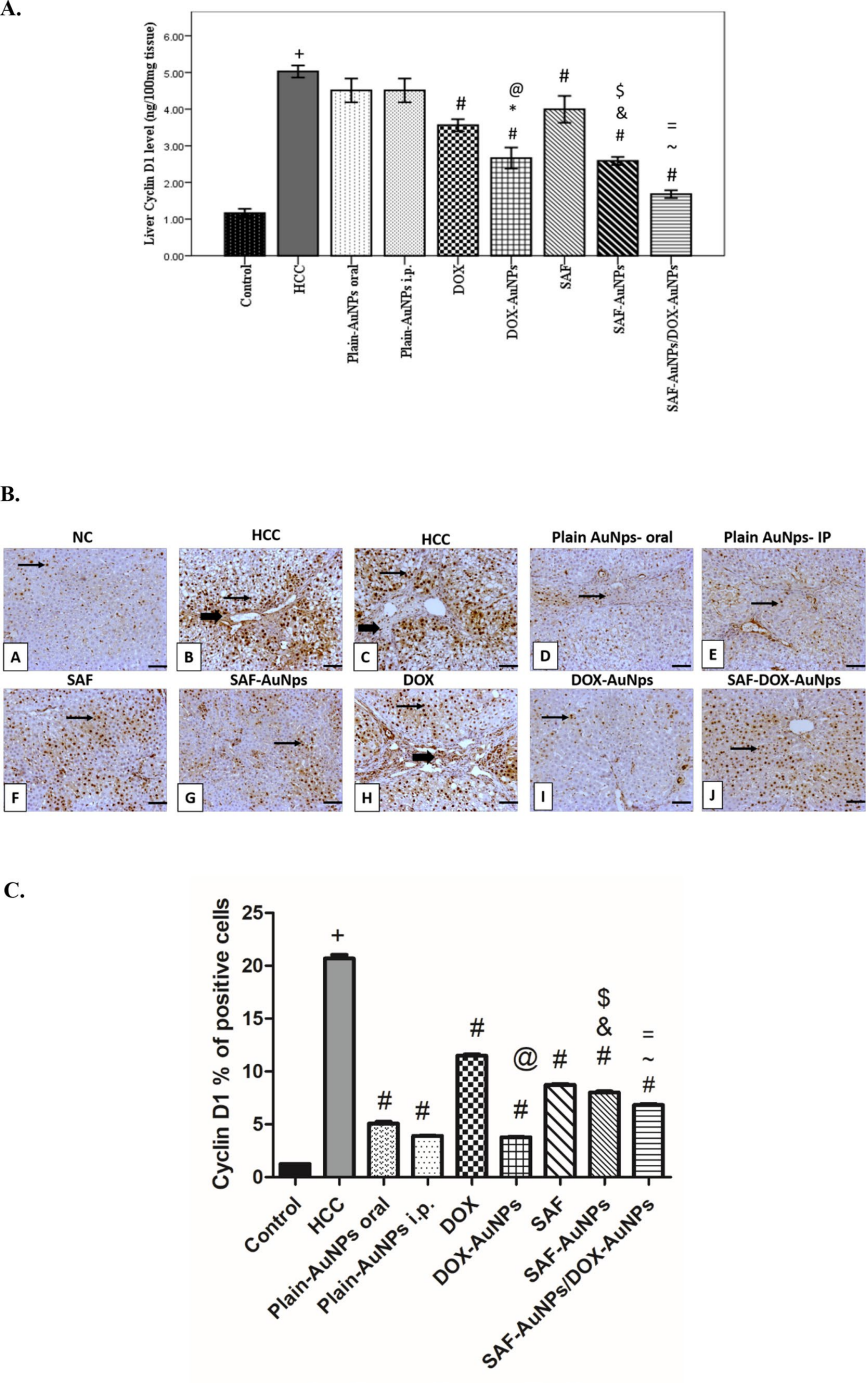
Effect of SAF-AuNPs/DOX-AuNPs combination therapy on hepatic Cyclin D1 protein level. **a** ELISA results showed that SAF-AuNPs/DOX-AuNPs combination significantly decreased hepatic cyclin D1 compared to HCC group. **b** Immunohistochemical-stained liver sections of Cyclin D1, black arrows indicate Cyclin D1 antibody-positive regions. **A** Control group showing faint immunopositive nuclear expression of cyclin D1 in hepatocytes. **B**, **C** HCC group showing diffuse high immunopositive nuclear stained hepatocytes with moderate stained inflammatory cells of the dense periportal bridging fibrosis that dividing hepatocytes into multiple nodules. **D** Plain AuNps oral group showing mild nuclear stained hepatocytes with few positivity in fibroblasts. **E** Plain AuNps- IP showing mild nuclear stained hepatocytes with mild positivity in periportal fibrosis. **F** SAF showing moderate nuclear expression in hepatocytes. **G** SAF- AuNps showing mild to moderate nuclear expression of cyclin D1 in hepatocytes. **H** DOX showing moderate to high expression in hepatocytes with nuclear expression in periportal bridging fibroblasts and inflammatory cells. **I** DOX- AuNps showing few nuclear positivity in hepatocytes. **J** SAF-DOX- AuNps showing moderate nuclear expression in hepatocytes. Thin arrow = positive hepatocytes, thick arrow = positive fibroblasts and inflammatory cells of fibrous septa. X100, bar = 100 µm. **c** Cyclin D1 percentage of positive cells in hepatic tissue. Results were presented as mean ± SEM. ^+^p < 0.05 vs. control group, ^#^p < 0.05 vs. HCC group, ^&^p < 0.05 vs. Plain-AuNPs oral group, ^$^p < 0.05 vs. SAF group ^@^p < 0.05 vs. DOX group, ^~^p < 0.05 vs. SAF-AuNPs group, ^=^p < 0.05 vs. DOX-AuNPs group

Hepatic Cyclin D1 protein level which was measured by ELISA showed a significant increase in the HCC group (p < 0.001) compared to the control group, whereas SAF and DOX groups significantly decreased hepatic Cyclin D1 level (p < 0.001) as compared to the HCC group. SAF-AuNPs group showed a significant decrease in hepatic Cyclin D1 level by 49.11% (p < 0.001) as compared to the HCC group, and by 35.09% (p < 0.001) as compared to the SAF group. DOX-AuNPs group showed a significant decrease in hepatic Cyclin D1 level by 47.12% (p < 0.001) as compared to the HCC group, and by 25.28% (p < 0.001) as compared to the DOX group. Also, SAF-AuNPs and DOX-AuNPs revealed a significant decrease in hepatic Cyclin D1 level as compared to Plain-AuNPs oral and Plain-AuNPs i.p. (p < 0.001). Additionally, SAF-AuNPs/DOX-AuNPs combination showed a significant reduction in hepatic Cyclin D1 level than HCC group (p < 0.001) and (p < 0.01) than SAF-AuNPs and DOX-AuNPs, respectively (Fig. 9a). Also, immunohistochemical analysis was conducted to evaluate Cyclin D1 expression in all studied groups. HCC group revealed a significant increase in the Cyclin D1 percentage of positive cells compared to the control group. Furthermore, the SAF-AuNPs group showed a considerable reduction in the Cyclin D1 percentage of positive cells than HCC and SAF groups. Also, the DOX-AuNPs group showed a significant reduction in the Cyclin D1 percentage of positive cells than HCC and DOX groups. In addition, SAF-AuNPs and DOX-AuNPs revealed a significant reduction in the Cyclin D1 percentage of positive cells than HCC group and either SAF-AuNPs or DOX-AuNPs groups (Fig. 9b, c).

### Effect of SAF-AuNPs/DOX-AuNPs combination therapy on hepatic MMP-9 protein level

**Fig. 10:**
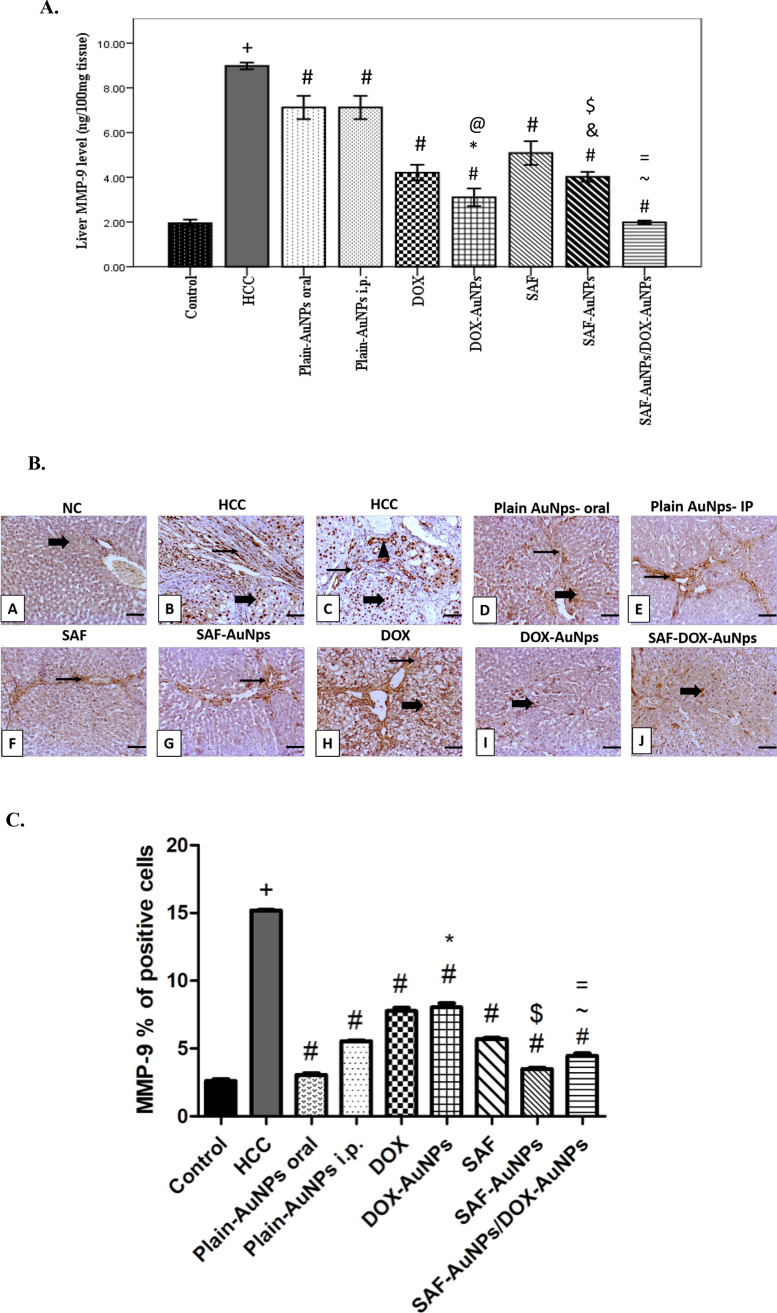
Effect of SAF-AuNPs/DOX-AuNPs combination therapy on hepatic MMP-9 protein level. **a** ELISA results showed that SAF-AuNPs/DOX-AuNPs combination significantly decreased hepatic MMP-9 compared to HCC group. **b** Representative IHC of MMP-9 expression in hepatic sections of different treatment groups. **A** Control group showing faint immunopositive cytoplasmic expression of MMP-9 in hepatocytes. **B**, **C** HCC group showing high immunopositive cytoplasmic and nuclear stained hepatocytes with extensive epithelial expression in newly formed bile ductules and fibroblasts. **D** Plain AuNps oral group showing mild cytoplasmic stained hepatocytes. **E** Plain AuNps- IP showing mild to moderate immunopositive cytoplasmic staining periportal fibrosis. **F** SAF showing mild to moderate expression in the thin fibrous septa that surrounded hepatocytes. **G** SAF- AuNps showing mild to moderate expression of MMP-9 in fibrous septa and admixed inflammatory cells. **H** DOX showing moderate to high expression in the vacuolated hepatocytes and the surrounding fibrous septa. **I** DOX-AuNps showing few scattered immunopositive stained hepatocytes. **J** SAF-DOX-AuNps showing mild to moderate cytoplasmic expression in hepatocytes. Thin arrow = positive fibrous septa, thick arrow = positive hepatocytes, arrowhead = positive biliary epithelial cells. Image magnification = 100x, scale bar = 100 μm. **c** MMP-9 percentage of positive cells in hepatic tissue. Results were presented as mean ± SEM. ^+^p < 0.05 vs. control group, ^#^p < 0.05 vs. HCC group, ^*^p < 0.05 vs. Plain-AuNPs i.p. group, ^$^p < 0.05 vs. SAF group, ^~^p < 0.05 vs. SAF-AuNPs group, ^=^p < 0.05 vs. DOX-AuNPs group

Hepatic MMP-9 protein level revealed a significant increase in the HCC group (p < 0.001) compared to the control group, whereas SAF and DOX groups significantly decreased hepatic MMP-9 level (p < 0.001) as compared to the HCC group. SAF-AuNPs group showed a significant decrease in hepatic MMP-9 level by 55.23% (p < 0.001) than HCC group, and by 20.87% (p < 0.01) than SAF group. DOX-AuNPs group showed extensive reduction in MMP-9 level by 65.48% (p < 0.001) as compared to the HCC group, and by 26.37% (p < 0.001) as compared to the DOX group. Also, SAF-AuNPs and DOX-AuNPs revealed a significant decrease in hepatic MMP-9 level as compared to Plain-AuNPs oral and Plain-AuNPs i.p. (p < 0.001). Moreover, SAF-AuNPs/DOX-AuNPs combination showed a significant decrease in hepatic MMP-9 level as compared to the HCC group (p < 0.001) and (p < 0.01) as compared to SAF-AuNPs and (p < 0.001) compared to DOX-AuNPs **(**Fig. 10a**)**. Also, immunohistochemical analysis was conducted to evaluate MMP-9 expression in all studied groups. HCC group revealed a significant increase in the MMP-9 percentage of positive cells compared to the control group. Furthermore, the SAF-AuNPs group showed considerable reduction in the MMP-9 percentage of positive cells than HCC or SAF group. Also, the DOX-AuNPs group showed a significant reduction in the MMP-9 percentage of positive cells than HCC group. In addition, SAF-AuNPs and DOX-AuNPs revealed a significant reduction in the MMP-9 percentage of positive cells than HCC group and either SAF-AuNPs or DOX-AuNPs groups (Fig. 10b, c).

### Effect of SAF-AuNPs/DOX-AuNPs combination therapy on hepatic VEGF

**Fig. 11:**
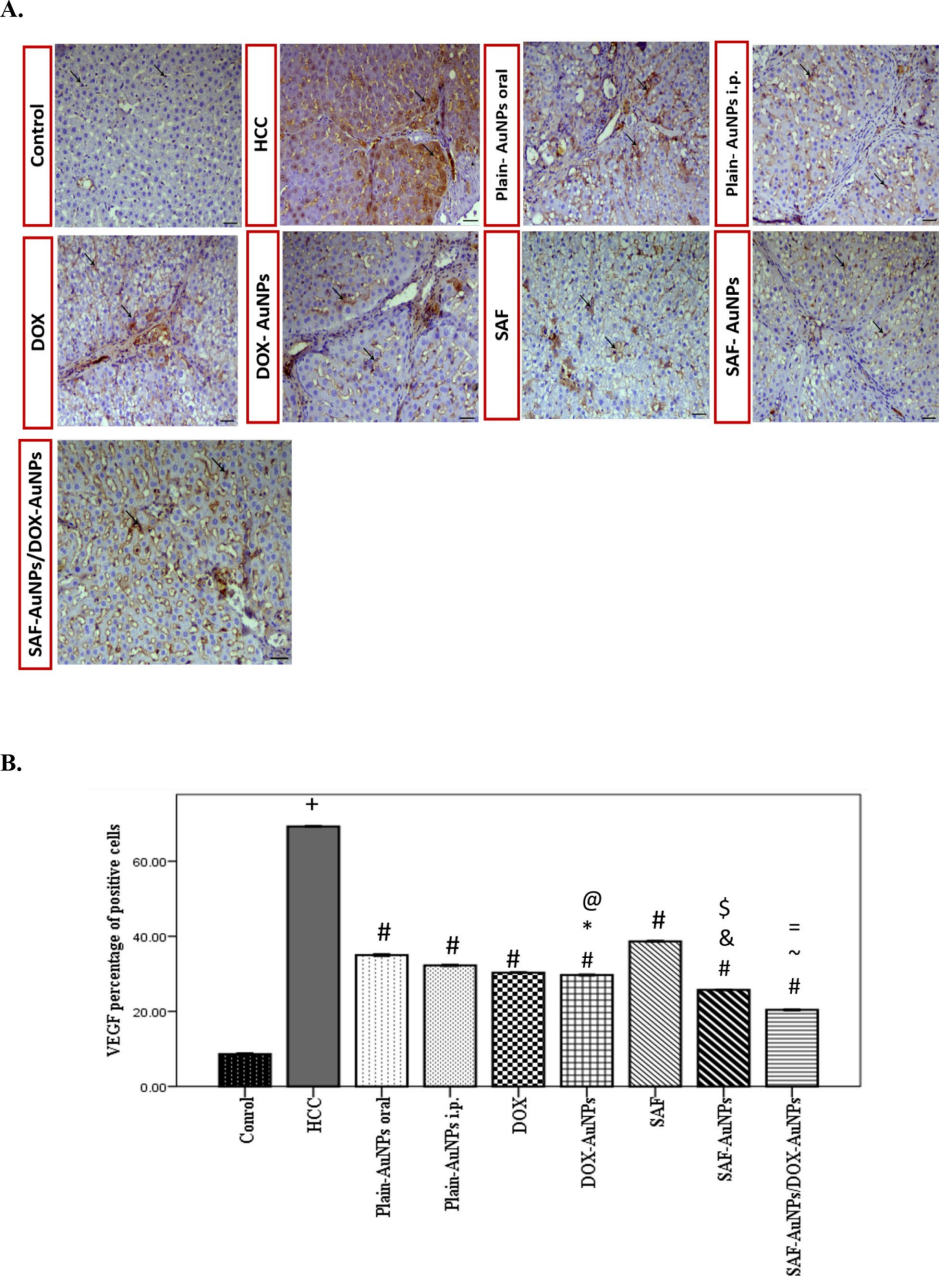
Effect of SAF-AuNPs/DOX-AuNPs combination therapy on hepatic VEGF expression. **a** Immunohistochemical-stained liver sections of VEGF, black arrows illustrate VEGF expression areas. X200, bar = 50 µm. **b** VEGF percentage of positive cells in hepatic tissue. Results were presented as mean ± SEM. n = 8 rats per group. ^+^p < 0.05 vs. control group, ^#^p < 0.05 vs. HCC group, ^*^p < 0.05 vs. Plain-AuNPs i.p. group, ^&^p < 0.05 vs. Plain-AuNPs oral group, ^$^p < 0.05 vs. SAF group ^@^p < 0.05 vs. DOX group, ^~^p < 0.05 vs. SAF-AuNPs group, ^=^p < 0.05 vs. DOX-AuNPs group

Immunohistochemical analysis was conducted to evaluate VEGF expression in all studied groups (Fig. 11a). The HCC group revealed a significant increase in the VEGF percentage of positive cells (p < 0.001) compared to the control group. Plain-AuNPs oral and Plain-AuNPs i.p. groups significantly reduced the VEGF percentage of positive cells (p < 0.001) than HCC group. Also, SAF and DOX groups highly decreased the VEGF expression (p < 0.001) than HCC group. Furthermore, the SAF-AuNPs group showed a considerable reduction in the VEGF percentage of positive cells than either HCC or SAF groups (p < 0.01). Also, the DOX-AuNPs group showed a significant reduction in the VEGF percentage of positive cells than HCC and DOX groups (p < 0.001). In addition, SAF-AuNPs and DOX-AuNPs revealed a significant reduction in the VEGF percentage of positive cells than Plain-AuNPs oral and Plain-AuNPs i.p. (p < 0.001). Moreover, SAF-AuNPs/DOX-AuNPs combination therapy revealed a significant reduction in the VEGF percentage of positive cells than HCC group (p < 0.001) and either SAF-AuNPs or DOX-AuNPs (p < 0.001) groups (Fig. 11b).

### Effect of SAF-AuNPs/DOX-AuNPs combination therapy on TAA-induced hepatic oxidative stress

**Fig. 12:**
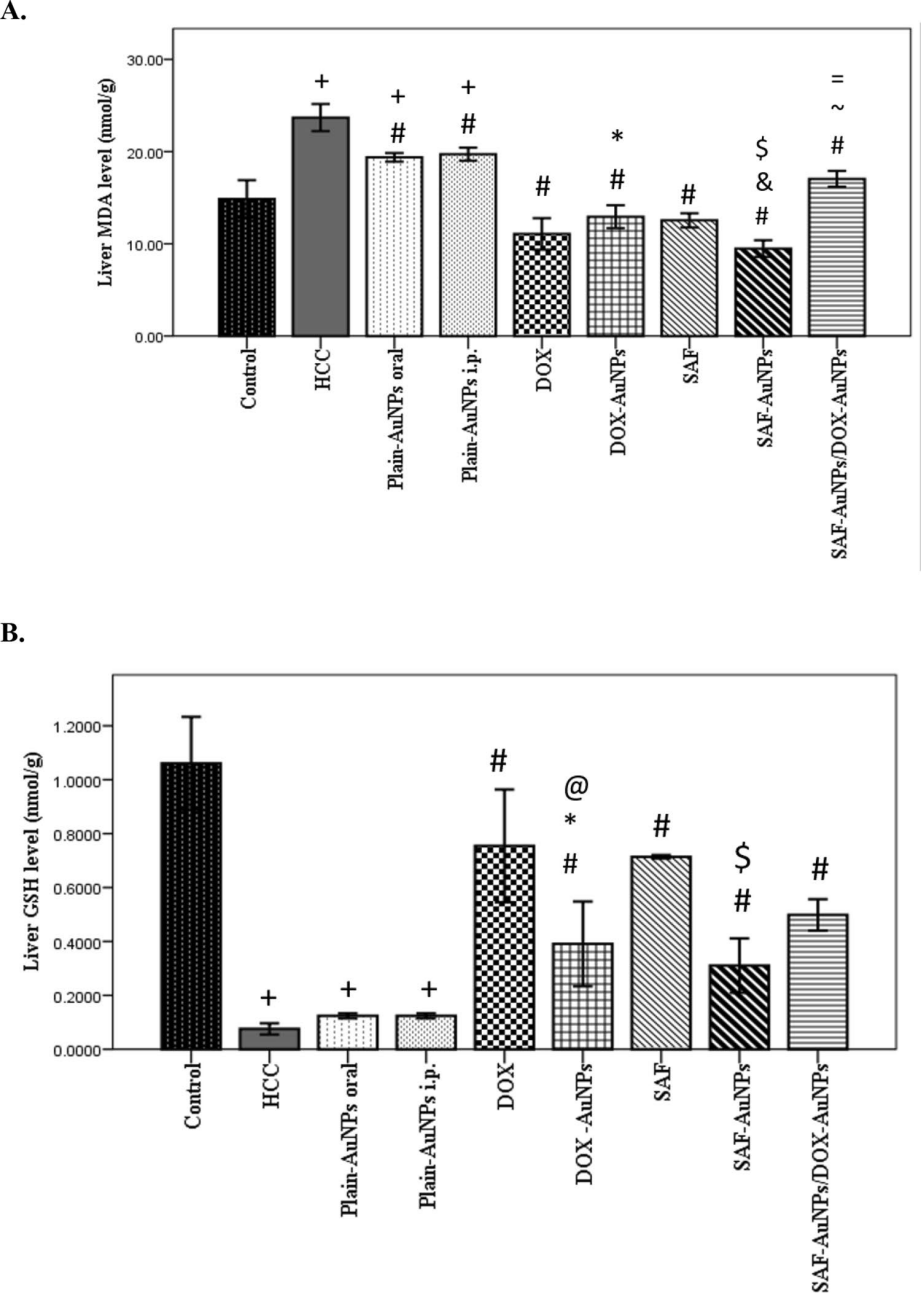
Effect of SAF-AuNPs/DOX-AuNPs combination therapy on TAA-induced hepatic oxidative stress parameters (**a**) Hepatic MDA content and (**b**) Hepatic GSH content. Results were presented as mean ± SEM. n = 8 rats per group. ^+^p < 0.05 vs. control group, ^#^p < 0.05 vs. HCC group, ^*^p < 0.05 vs. Plain-AuNPs i.p. group, ^&^p < 0.05 vs. Plain- AuNPs oral group, ^$^p < 0.05 vs. SAF group, ^@^p < 0.05 vs. DOX group, ^~^p < 0.05 vs. SAF-AuNPs group, ^=^p < 0.05 vs. DOX-AuNPs group

The TAA-induced HCC group revealed a significant increase in MDA level in the liver than control group (p < 0.001). Plain-AuNPs oral and i.p. groups showed a significant rise in hepatic MDA level than control group (p < 0.001) but revealed a significant decline in hepatic MDA level than HCC group (p < 0.001). In addition, SAF and DOX groups significantly decreased hepatic MDA level (p < 0.001) as compared to the HCC group. SAF-AuNPs group showed a significant decrease in hepatic MDA level by 59.97% (p < 0.001) and by 24.4% (p < 0.001) compared to HCC and SAF groups, respectively. Also, the DOX-AuNPs group significantly reduced hepatic MDA level by 45.4% (p < 0.001) as compared to the HCC group, but a non-significant increase when compared to the DOX group. SAF-AuNPs and DOX-AuNPs revealed a significant decrease in hepatic MDA level as compared to Plain-AuNPs oral and Plain-AuNPs i.p. (p < 0.001). Moreover, SAF-AuNPs/DOX-AuNPs combination therapy revealed a significant reduction in MDA level by 28.04% (p < 0.001) as compared to the HCC group but revealed a significant increase in hepatic MDA level (p < 0.001) when compared to either SAF-AuNPs or DOX-AuNPs (Fig. 12a).

On the flip side, the HCC group revealed a significant reduction in hepatic GSH level as compared to the control group (p < 0.001). Plain-AuNPs oral and i.p. groups showed a significant decrease in hepatic GSH level compared to the control group (p < 0.001), but a non-significant increase in GSH level compared to the HCC group. SAF and DOX groups significantly increased hepatic GSH level (p < 0.001) as compared to the HCC group. Furthermore, the SAF-AuNPs group showed a significant rise in GSH level (p < 0.01) compared to the HCC group and showed a significant reduction in GSH level (p < 0.001) as compared to the SAF group. DOX-AuNPs group showed a significant increase in hepatic GSH level (p < 0.001) as compared to the HCC group, with a significant decrease in GSH level (p < 0.001) compared to the DOX group. Also, DOX-AuNPs showed a significant increase in hepatic GSH level more than Plain-AuNPs i.p. (p < 0.001). Moreover, SAF-AuNPs/DOX-AuNPs combination therapy revealed a significant rise in hepatic GSH level (p < 0.001) as compared to the HCC group but revealed a non-significant increase in hepatic GSH level compared to either SAF-AuNPs or DOX-AuNPs (Fig. 12b).

## Discussion

Liver transplantation has provided survival benefits to patients with localized HCC. However, diagnosis of many cases at advanced stages, limited availability of donor livers, and improved mortality for patients without transplants have limited the impact of transplantation on general population HCC-specific mortality rates [31]. Thus, many researchers have been focusing on developing new effective therapeutic approaches for HCC to decrease mortality and improve survival rates. In context, the current study studied the efficacy of SAF either alone or loaded on AuNPs (SAF-AuNPs) as a treatment for TAA-induced HCC in rats. Also, to overcome DOX chemo-resistance in HCC and increase its therapeutic effect we examined the efficacy of DOX and DOX-AuNPs in the treatment of HCC. Moreover, our study showed a marked therapeutic efficacy of SAF-AuNPs/DOX-AuNPs combination in the treatment of 8HCC in rats.

HCC induction in rats by TAA at the chosen dose was confirmed by the significant increase in AFP and disrupted parenchymal structure in histopathological examination. SAF showed potent anti-inflammatory, antioxidant and anti-cancer activities [15, 32]; and has the ability to induce apoptosis in various cancer cell lines [33]. AuNPs target the drug to the cancer sites as they can pass through physiological barriers and passively accumulate within tumor sites thus increasing drugs’ therapeutic activity and decreasing their side effects (Supplementary Data). AuNPs efficiently bind to target cells with high avidity, this usually results in improved cellular uptake [21]. Gold nanoparticles are promising for various biomedical uses, including drug delivery, medical imaging, and photothermal therapy. However, their clinical application faces limitations such as potential toxicity, inefficient clearance from the body, stability issues, and high production costs. Current research focuses on improving their design through innovations like biodegradable gold clusters and enhanced surface modifications to overcome these limitations and facilitate their widespread adoption in medicine [34].

Therefore, we were inspired in the present study to evaluate the effect of SAF, SAF-AuNPs, and a combination of SAF-AuNPs and DOX-AuNPs in the HCC rat model. Our results showed that SAF enhanced liver functions and reduced fibrosis and necro-inflammation. Also, the SAF group showed improvement in hepatic parenchymal structure and a significant decrease in serum AFP. For SAF-AuNPs and DOX-AuNPs, our transmission electron microscopic (TEM) images showed an increase in nanoparticle size indicating some type of attachment between SAF and DOX and prepared AuNPs. Also, a change in surface morphology can be attributed to the coating process where AuNPs act as a nucleating agent for a crystalline structure as revealed from the electron diffraction pattern (Fig. 5S and Fig. 6S).

AuNPs enhanced SAF and DOX antitumor activity as indicated by the significant decrease in serum AFP in SAF-AuNPs and DOX-AuNPs groups than SAF and DOX groups, respectively. More interestingly, examination of hepatic tissues showed a marked enhancement in SAF-AuNPs/DOX-AuNPs group than each one alone.

Wnt/β-catenin pathway is commonly activated in HCC [4]. Apparent activation of Wnt-3a in the canonical pathway was associated with HCC incidence and progression [35]. Hepatic Wnt-3a expression was significantly increased in HCC, with gradually up-regulating its protein level in liver tissues, indicating that Wnt-3a might participate in promoting tumorigenesis of HCC [36]. However, limited data are available on the underlying mechanisms of Wnt-3a during hepato-carcinogenesis and therapeutic targeting value [35, 36]. Consistently, the current study showed that hepatic Wnt-3a level was significantly elevated in the TAA-induced HCC group. To the best of our knowledge, this study is the first study to signify the antitumor effect of SAF through down-regulation of the Wnt/β-catenin signaling pathway. SAF significantly decreased Wnt-3a level in the liver more than the HCC group. In addition, AuNPs enhanced SAF and DOX antitumor activity as showed by the significant decrease in hepatic Wnt-3a level in SAF-AuNPs and DOX-AuNPs groups as compared to SAF and DOX groups, respectively.

Binding of Wnt-3a to Frizzled receptor and LRP5/6 co-receptors results in the recruitment of Dvl and destruction complex to the cell membrane, leading to dissociation of the destruction complex and finally stabilization and accumulation of β-catenin in the cytoplasm, which translocates to the nucleus and induces target gene expression. In accordance with our results, the hepatic β-catenin protein level was significantly elevated in the HCC group compared with the control group. This elevation of hepatic β-catenin protein level is possibly attributed to Wnt-3a elevation which leads to aberrant activation of Wnt/β-catenin signaling and finally, accumulation of β-catenin in the cytoplasm and nucleus. A recent study showed that overexpression of hepatic β-catenin in patients with HCC has a poor prognosis with a lower survival rate [37].

As we mentioned before SAF significantly decreased hepatic Wnt-3a level and this effect is accompanied by a decrease in total β-catenin protein level. Moreover, AuNPs enhanced SAF and DOX effect on β-catenin as determined by the significant decrease in hepatic β-catenin level in SAF-AuNPs and DOX-AuNPs groups as compared to SAF and DOX groups, respectively. More interestingly, hepatic β-catenin level showed a marked decrease in SAF-AuNPs/DOX-AuNPs combination group that exceeds each treatment alone. Thus, the overall effect of our therapy on β-catenin may suggest that without enough Wnt3a activation, β-catenin may no longer be rescued from its fate by the destruction complex. MDR protein is an important target of β-catenin and a known mediator of chemo-resistance [38].

A previous study has indicated that inhibition of Wnt⁄β-catenin signaling down-regulates MDR protein and reverses MDR of cholangiocarcinoma [39]. Hence, the Wnt⁄β-catenin pathway might be an ideal chemo-sensitization target, our study revealed that AuNPs markedly decreased hepatic MDR level when combined with SAF and DOX as compared with either treatment alone. In addition, a significant lowering effect in hepatic MDR level was observed in SAF-AuNPs/DOX-AuNPs combination therapy when compared to each group alone.

Furthermore, Cyclin D1 is another important downstream target of β-catenin and plays an important role in cell cycle progression [38]. Cyclin D1 overexpression may be an early event during hepatocarcinogenesis and play a role in HCC differentiation [40]. Our study revealed that in the HCC group hepatic Cyclin D1 level was significantly elevated. On the other hand, SAF significantly decreased hepatic Cyclin D1 level and its apoptotic and antiproliferative effect was markedly potentiated by AuNPs. Moreover, SAF-AuNPs/DOX-AuNPs combination therapy showed a remarkable decrease in hepatic Cyclin D1 level.

Additionally, angiogenic factors; VEGF and MMP-9 are also activated by β-catenin and play an important role in the regulation of HCC angiogenesis and metastasis [2, 41]. Likewise, our study revealed that both hepatic MMP-9 level and VEGF expression were significantly elevated in the HCC group. Interestingly, SAF suppressed VEGF expression and MMP-9 level significantly and the antiangiogenic effect of SAF was markedly increased when combined with AuNPs. Moreover, SAF-AuNPs/DOX-AuNPs combination therapy showed a remarkable decrease in hepatic MMP-9 level and VEGF expression. Thus, SAF and SAF-AuNPs could potentially inhibit Wnt⁄β-catenin signaling in TAA-induced HCC in rats.

One of the causes behind the hepatocyte injury is TAA-induced hepatic oxidative stress [42]. Our data showed that liver cells were exposed to oxidative stress as indicated by elevated hepatic MDA level in the HCC group indicating a marked lipid peroxidation status. Moreover, the antioxidant defense system was diminished as indicated by the decreased hepatic GSH level. Although SAF-AuNPs and DOX-AuNPs enhanced liver function and showed significant antitumor activity, they produced a significant decrease in GSH level. Also, SAF-AuNPs/DOX-AuNPs combination therapy showed an increase in MDA level compared to either SAF-AuNPs or DOX-AuNPs, despite the pronounced anticancer activity of SAF-AuNPs/DOX-AuNPs combination therapy which exceeds each treatment alone. This may relate to the oxidative stress effect of AuNPs as a mechanism to induce cytotoxicity in tumor cells [43, 44]. On the contrary, SAF treatment decreased oxidative stress via lowering MDA and elevating GSH levels. The established antioxidant capacity of SAF agreed with a previous study [27].

Recent studies have demonstrated that AuNPs have been used for the treatment of several tumors like papillary thyroid carcinoma [45] and colon cancer [46]. These recent studies emphasized that the antitumor effect of AuNPs was related to its antiangiogenic properties and its cytotoxic oxidative stress effect. In context, our study revealed that AuNPs may have an antitumor effect against HCC. Plain-AuNPs significantly decreased fibrosis percentage, hepatic Wnt-3a, β-catenin, and MMP-9 levels, and VEGF expression within the hepatic tissue when compared to HCC. Moreover, Plain-AuNPs showed a significant decrease in hepatic GSH level and significant elevation in hepatic MDA level compared to the control group.

## Conclusion

SAF has a hopeful antitumor activity for the treatment of HCC which is primarily related to marked attenuation of the Wnt/β-catenin signaling pathway, which in turn down-regulates the cell cycle and tumor angiogenesis. Developing SAF-AuNPs showed enhanced anticancer activity in the treatment of HCC compared to SAF alone, possibly due to AuNP’s passive targeting ability to the tumor site. Moreover, SAF-AuNPs enhanced DOX-AuNPs antitumor activity and attenuated DOX chemo-resistance. So, the desired therapeutic effect may be obtained with minor doses and lowering the associated side effects. Finally, the current study suggests that SAF and the newly developed SAF-AuNPs have a promising therapeutic effect that can be used in the treatment of HCC.

## Supplementary Information

Supplementary material 1.

## Data Availability

“The authors declare that the data supporting the findings of this study are available within the paper and its Supplementary Information files. Should any raw data files be needed in another format they are available from the corresponding author upon reasonable request”.

## Abbreviations

ALP: Alkaline phosphatase
ALT: Alanine aminotransferase
AST: Aspartate aminotransferase
AuNPs: Gold nanoparticles
CK1: Casein kinase 1
DAB: Diaminobenzidine
DOX: Doxorubicin
AFP: Alpha-fetoprotein
ELISA: Enzyme-linked immunosorbent assay
EPR: Enhanced permeability and retention
FZ: Frizzled; GGT: Gamma-glutamyl transferase
GSH: Glutathione
GSK: Glycogen synthase kinase
H&E: Hematoxylin and eosin
HAI: Histology activity index
HCC: Hepatocellular carcinoma
i.p.: Intra-peritoneal
MDA: Malondialdehyde
MDR: Multi-drug resistance
MMP-9: Matrix metalloproteinase-9
PBS: Phosphate buffered saline
SAF: Safranal
SAED: Selected area electron diffraction
TAA: Thioacetamide
TEM: Transmission electron microscope
UV–vis: Ultraviolet–visible
VEGF: Vascular endothelial growth factor
