# Supplementary material for: Safranal-loaded gold nanoparticles alleviate hepatocellular carcinoma via targeting the Wnt/β-catenin pathway
Source: Discov Oncol. 2025 May 19;16:821. doi: 10.1007/s12672-025-02447-w (PMC12089598; doi:10.1007/s12672-025-02447-w)
Supplement: Supplementary file 1 — Supplementary material 1. [file 12672_2025_2447_MOESM1_ESM.docx]

**Nanoparticle characterization**

**1. AuNP characterization**

Ultraviolet-visible (UV-vis) spectroscopy is considered as a main, fast and effective tool that can be used for the identification of nanoparticle formation and stability in liquids. Green synthesized gold nanoparticles (AuNPs) can be easily observed through characteristic color solution of colloidal gold assigned to the surface plasmon resonance (SPR) results from the oscillations of free conduction electrons convinced with interaction with electromagnetic fields. Obtained spectral data characterized by two main absorption bands centered at 210 and 533 nm **(Fig. 1S)**. Strong UV absorption band at 210 nm correlated with the residuals of plant extract used for synthesis of gold even present in ppm level, while the strong visible band centered at about 533 nm is known to be characterize the SPR process of synthesized AuNPs.

**Figure 1S:** UV-vis spectroscopy of gold nanoparticles. Obtained spectral data characterized by two main absorption bands centered at 210 and 533 nm. Strong UV absorption band at 210 nm correlated with the residual of plant extract used for synthesis of gold even present in ppm level, while the strong visible band centered at about 533 nm is known to be characterize the surface plasmon resonance (SPR) process of synthesized gold nanoparticles (AuNPs).

Crystalline nature of the synthesized AuNPs may be observed through the appearance of characteristic sharp bands in the X-ray diffraction (XRD) Bragg reflections originally suited at angles 38.5^°^, 44.0^°^, 64.2^°^, 77.5^°^ and 81.5^°^ usually correlated and assigned to (1 1 1), (2 0 0), (2 2 0), (3 1 1), and (2 2 2) reflection planes of face-centered cubic (*fcc*) crystal structure. These findings agree with the diffraction peaks corresponding to *fcc* structure of gold standard (JCPDS card no. 04-0784) **(Fig. 2S)**.

**Figure 2S:** X-ray diffraction (XRD) analysis of gold nanoparticles. Appearance of characteristic sharp bands originally suited at angles 38.5°, 44.0°, 64.2°, 77.5° and 81.5° usually correlated and assigned to (1 1 1), (2 0 0), (2 2 0), (3 1 1), and (2 2 2) reflection planes of face-centered cubic (*fcc*) crystal structure.

Transmission electron microscopic (TEM) investigation of synthesized AuNPs reveals a uniform distribution of distorted spherical nanoparticles of average size ranging between 9 – 18 nm. The diffraction pattern is with agreement with the results retained from XRD **(Fig. 3S)**.


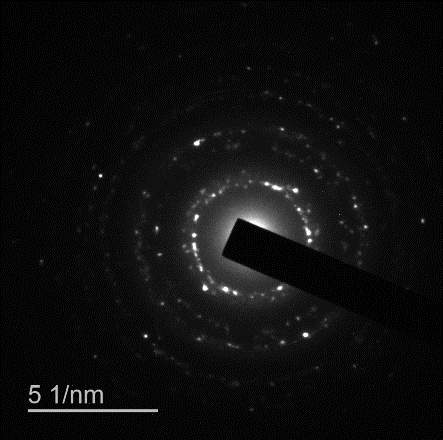

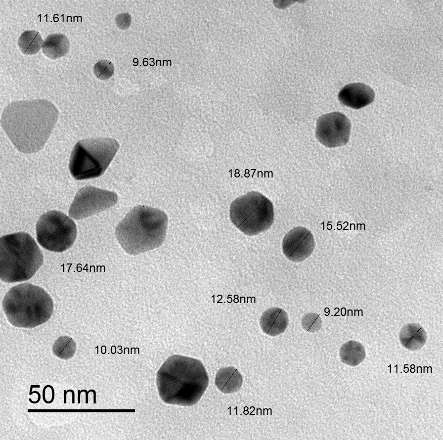

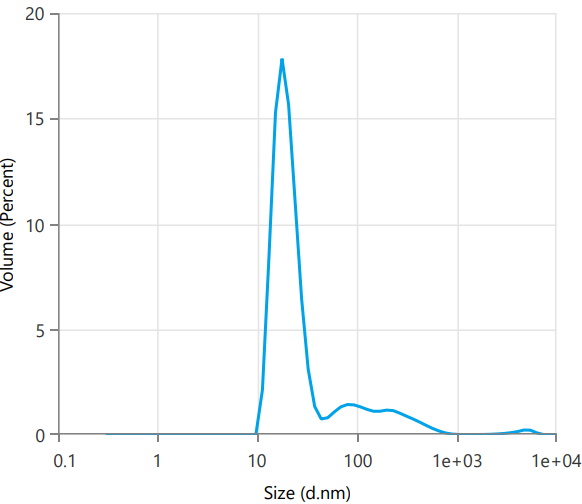


**Figure 3S:** Transmission electron microscopic (TEM) images of gold nanoparticles with DLS. Uniform distribution of distorted spherical nanoparticles of average size ranging between 9 – 18 nm.

A


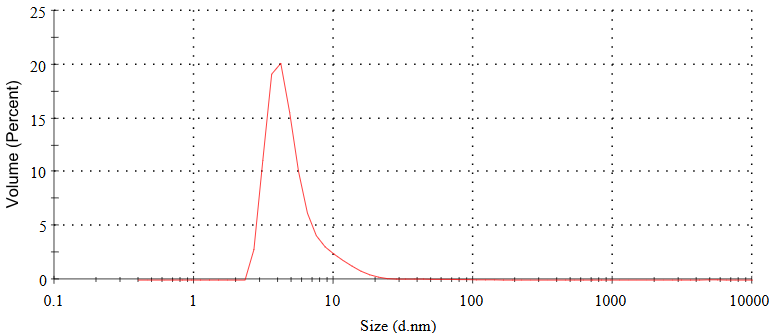


B

**
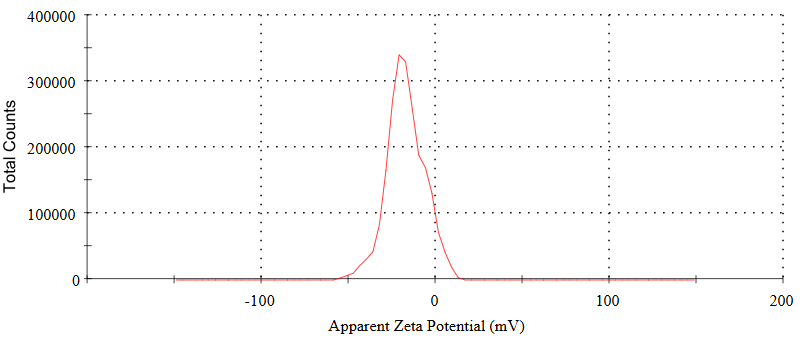
**

**Figure 4S:** Zeta size (A) and potential (B) of gold nanoparticles.

**2. SAF-AuNPs and Dox-AuNPs characterization**

Both transmission electron micrographs of Safranal (SAF)-AuNPs and Doxorubicin (DOX)-AuNPs reveal an increase of nanoparticle size in both cases indicating some type of attachment between drug and prepared AuNPs. Besides, a change in surface morphology can be attributed to coating process where AuNPs acting as a nucleating agent for a crystalline structure as reveled from the electron diffraction pattern in both cases **(Fig. 5S and Fig. 6S)**.


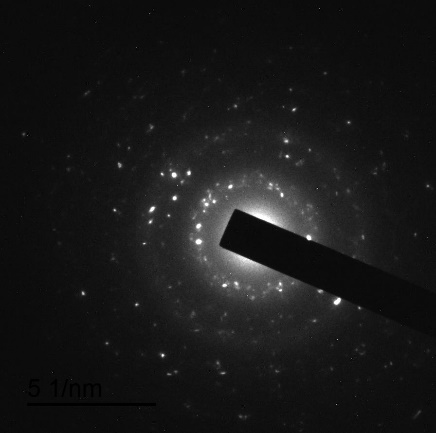

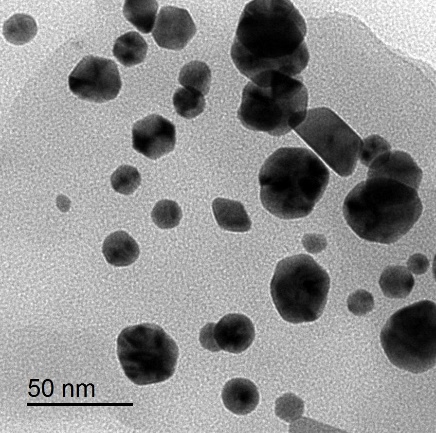


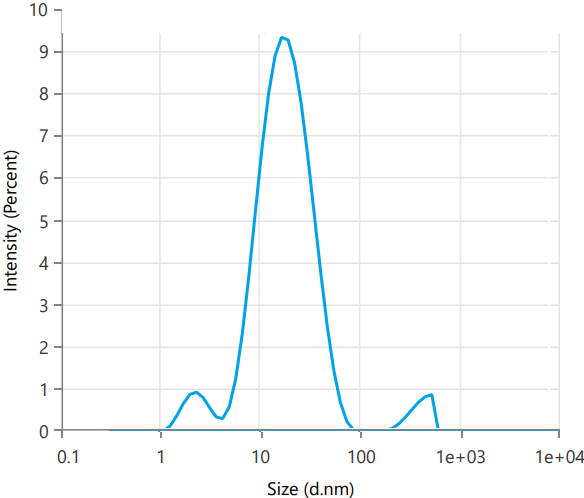


**Figure 5S:** Transmission electron microscopic (TEM) images of SAF-AuNPs. Increase of nanoparticle size indicating some type of attachment between SAF and prepared AuNPs. Also, a change in surface morphology can be attributed to coating process where AuNPs acting as a nucleating agent for a crystalline structure as reveled from the electron diffraction pattern.


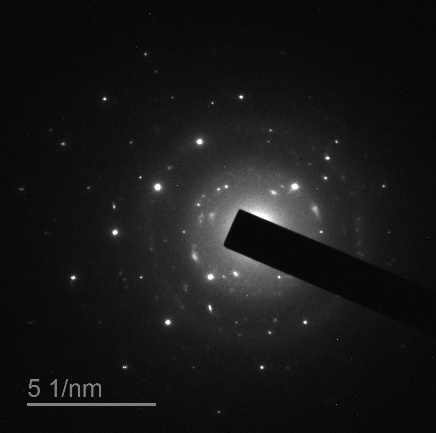

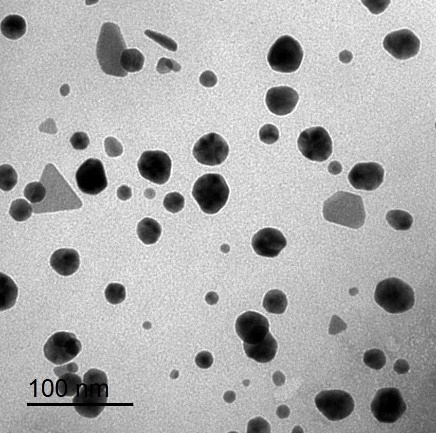

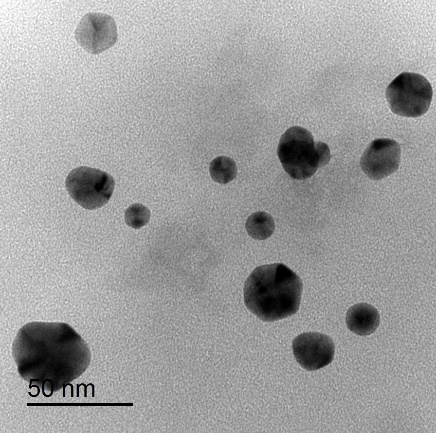

**Figure 6S:** Transmission electron microscopic (TEM) images of DOX-AuNPs. Increase of nanoparticle size indicating some type of attachment between DOX and prepared AuNPs. Also, a change in surface morphology can be attributed to coating process where AuNPs acting as a nucleating agent for a crystalline structure as reveled from the electron diffraction pattern.
